# Association of Agronomic Traits with SNP Markers in Durum Wheat (*Triticum turgidum* L. *durum* (Desf.))

**DOI:** 10.1371/journal.pone.0130854

**Published:** 2015-06-25

**Authors:** Xin Hu, Jing Ren, Xifeng Ren, Sisi Huang, Salih A. I. Sabiel, Mingcheng Luo, Eviatar Nevo, Chunjie Fu, Junhua Peng, Dongfa Sun

**Affiliations:** 1 College of Plant Science and Technology, Huazhong Agricultural University, Wuhan Hubei, 430070, China; 2 Shandong Provincial Key Laboratory of Functional Macromolecular Biophysics, Institute of Biophysics, Dezhou University, Dezhou, Shandong, 253023, China; 3 Department of Plant Sciences, University of California Davis, Davis, CA, 95616, United States of America; 4 Institute of Evolution, University of Haifa, Mount Carmel, Haifa, 31905, Israel; 5 Science and Technology Center, China National Seed Group Co., Ltd, Wuhan, Hubei, 430206, China; 6 Hubei Collaborative Innovation Center for Grain Industry, Jingzhou, Hubei, 434025, China; Murdoch University, AUSTRALIA

## Abstract

Association mapping is a powerful approach to detect associations between traits of interest and genetic markers based on linkage disequilibrium (LD) in molecular plant breeding. In this study, 150 accessions of worldwide originated durum wheat germplasm (*Triticum turgidum* spp. *durum*) were genotyped using 1,366 SNP markers. The extent of LD on each chromosome was evaluated. Association of single nucleotide polymorphisms (SNP) markers with ten agronomic traits measured in four consecutive years was analyzed under a mix linear model (MLM). Two hundred and one significant association pairs were detected in the four years. Several markers were associated with one trait, and also some markers were associated with multiple traits. Some of the associated markers were in agreement with previous quantitative trait loci (QTL) analyses. The function and homology analyses of the corresponding ESTs of some SNP markers could explain many of the associations for plant height, length of main spike, number of spikelets on main spike, grain number per plant, and 1000-grain weight, etc. The SNP associations for the observed traits are generally clustered in specific chromosome regions of the wheat genome, mainly in 2A, 5A, 6A, 7A, 1B, and 6B chromosomes. This study demonstrates that association mapping can complement and enhance previous QTL analyses and provide additional information for marker-assisted selection.

## Introduction

Durum wheat (*Triticum durum* Desf.) is a tetraploid species consisting of A and B genomes (AABB). It was resulted from domestication of wild emmer wheat (*T*. *dicoccoides*) derived from a spontaneous cross between *T*. *urartu* (AA genome, 2n = 14) and an ancient relative of *Aegilops speltoides* (donor of the BB genome) [1]. As the main source of semolina for the production of pasta, bagel, couscous and other Mediterranean local end-products [2], durum wheat is cultivated on about 17 million hectares worldwide. The durum wheat is mainly grown in Europe, Canada, Syria, USA, Algeria and Morocco, particularly in the Mediterranean, while minor grown in Russia, Turkey, Tunisia, Mexico and India [3]. It plays an important role in food production of these regions (http://www.pasta-unafpa.org/ingstatistics5.htm).

The amount of genetic variation in germplasm and genetic relationships between genotypes are very valuable information for effective conservation and utilization of genetic resources [4]. Genetic diversity is the foundation for survival, adaptation and evolution in time and space [5]. More knowledge on the genetic variation and the genetic determinants of diversity is useful for discovering new genes [6–9]. Its preservation and wise application in nature are the central aspect of biological conservation and genetic improvement. Assessment of genetic diversity in durum germplasm will provide useful information for breeding programs. Genetic diversity in germplasm can be characterized by different markers: like morphology, pedigree and molecular markers. Currently, application of molecular markers is the most effective and feasible method for characterizing diversity in wild and cultivated germplasm [7, 8].

Genetic diversity analysis of wild and cultivated wheat is generally based on low-to medium-throughput marker platforms such as restriction fragment length polymorphism (RFLP), random amplified polymorphic DNA (RAPD), amplified fragment length polymorphism (AFLP) and simple sequence repeat (SSR) [10–14]. These molecular markers have been shown to be useful for studying genetic diversity and structure, and differentiating durum wheat cultivars to some extent. However, these markers, especially RFLP, RAPD and AFLP, have not been used extensively in breeding programs because they are not more efficient for application in marker-assisted-selection (MAS) [15].

Single nucleotide polymorphism (SNP) can be converted into genetic markers amenable to high-throughput assays [16, 17]. As the continuous discovery of SNP and the further development of SNP-genotyping platforms, SNP markers gain increasing more and more attentions [18–21]. Genome-wide maps consisting of large number of SNP markers have been reported in *Arabidopsis* [22], rice [23], soybean [24] and barley [25]. However, large-scale SNP detection is restricted by both the polyploidy nature and the high sequence similarity among the three homoeologous genomes in wheat [26, 27]. A relatively small number of SNP markers are now available in wheat due to the genome complexity [18, 28].

Currently, SNP marker is the major type of molecular markers used in evaluating genetic diversity, population structure, familial kinship and associations in multiple organisms. The availability of wheat SNP markers allows trait-marker association analysis with a high efficiency in durum wheat. Association analysis, or association mapping (AM), is a method based on linkage disequilibrium (LD) that is used to detect the relationship between phenotypic variation and genetic polymorphisms [29, 30]. Originally developed for human genetics [31, 32], AM has recently appeared as an alternative approach to mapping QTLs and genes in many crops, due to the development of cheaper, faster and higher density molecular markers [33]. In comparison with genetic linkage analysis, AM has three obvious advantages that include shorter research time, much higher mapping resolution and a greater number of alleles [34]. Relative to other experimental designs that require sampling within families, AM offers the important advantage that allows sampling unrelated individuals in the population for studying genetics of complex traits [29, 35]. AM has been applied to many crop species, such as maize, soybean, rice, barley, wheat etc. [9, 36–38]. Therefore, AM provides a powerful tool for investigating genetics of quantitative traits in plant species [34, 39, 40].

Genetic diversity and association analysis in wheat germplasm have been studied using several types of molecular markers including SNP [9]. Recently, there are more reports on diversity pattern and population structure in durum wheat germplasm [41–43]. However, there are few reports published on trait-marker associations in durum wheat. Therefore, the major objective of this study is to reveal associations between quantitative traits and the SNP markers in durum wheat.

## Materials and Methods

### Plant materials and field trials

One hundred and fifty durum wheat accessions of worldwide origin were investigated in this study. The collection of the durum wheat germplasm was classified into seven groups based on their geographic origins. Of the accessions, 24 originated from West Asia (WA), 25 from East Asia (EA), 33 from North America (NA), 33 from different parts of Europe (EU), 12 from South America (SA), 16 from North Africa (AF), and 7 from Australia (AU). The name, place of origin and identifier number for each accession is listed in S1 Table.

In order to obtain reliable phenotypic data, field trials of all the accessions with replications were conducted in four consecutive years. The field trials got the approval of Huazhong Agricultural University, and were performed on the experimental farm of Huazhong Agricultural University, Wuhan, China. The land accessed is not privately owned nor protected, which is belong to Huazhong Agricultural University. All of the materials used in this study were acquired by Dr. Junhua Peng from USDA (United States Department of Agriculture), and no any protected species were sampled in the field trials. The trials with three replications were planted around the end of October in 2009, 2010, 2011 and 2012, respectively, in two rows with 1 m in length and 20 cm between rows, 6 plants in each row. Because some of the accessions were very tall and easy-lodging, we installed frames made of bamboo sticks in each plot before heading to prevent lodging, or reduce lodging impact on the traits.

### Phenotyping of the key traits

#### Measurement of key traits

After full maturity, we randomly harvested four individual plants from each plot. The following 10 traits were measured. The mean value of a trait in each replication was calculated.

PH: plant height (cm),
ES: number of effective spikes,
LMS: length of main spike (cm),
SMS: number of spikelets on main spike,
RLMS: rachis internode length of main spike (cm),
NSPP: number of spikelets per plant,
LFPMS: panicle neck length of main spike (cm),
GNP: grain number per plant,
GWP: grain weight per plant (g),
KGW: 1000-grain weight (g).

#### Variation analysis

The mean phenotypic values of the 10 quantitative traits were subjected to statistical analysis. Frequency distribution of the traits was analyzed, and Kolmogorov–Smirnov test was performed to test for normal distribution. Data transformation is performed for the traits that did not fit the normal distribution. Calculations of the descriptive statistics, analysis of variance (ANOVA) and broad-sense heritability (*H* ^*2*^), and correlation analysis were performed using SPSS programs (IBM SPSS Statistics, Chicago, IL, USA).

### DNA extraction, SNP genotyping and marker data analyses

Before the elongation stage of wheat plants, approximately 1.0 g of young leaf tissue was collected from each of the accessions. The tissue was placed in a 1.5 ml Eppendorf tube, immediately frozen in liquid N, and stored in a -80°C freezer [43]. The cetyltrimethyl ammonium bromide (CTAB) method was used to extract the total genomic DNA [44].

The DNA samples were shipped to University of California at Davis, USA for genotyping. A set of 1,536 genome-specific SNP markers were applied to genotype the germplasm. These SNP markers were discovered in a panel of 32 lines of tetraploid and hexaploid wheat (http://avena.pw.usda.gov/SNP/internal/protocol/id.htm), and downloaded from the Wheat SNP Database (http://probes.pw.usda.gov:8080/snpworld/Search). The SNP-genotyping was performed using the Illumina Bead Array platform and Golden Gate Assay (Illumina, San Diego, CA) at the UC Davis Genome Center (http://www.genomecenter.ucdavis.edu/dna_technologies). The SNP markers were treated as co-dominant markers. The details of genotyping and genetic analyses were described in Ren et al. [43].

### Linkage disequilibrium

It is essential for association mapping to examine the degree of LD in the genome and chromosome [45,46]. The fraction of locus pairs indicating significant LD increases with decreasing significance level. A high significance level of p<0.001 was chosen for comparative purposes. If all pairs of adjacent loci within a chromosomal region were in significant LD, this region was treated as a LD block [47]. LD between markers was measured using R^2^, square of correlation between the markers [48]. The values of R^2^ and P were calculated using the software TASSEL 3.0.124 (http://www.maizegenetics.net/).

### Association analysis

Association mapping analysis between SNP markers and the 10 quantitative traits (PH, ES, LMS, SMS, RLMS, LFPMS, NSPP, GNP, GWP, and KGW) was performed based on the general linear model (GLM) and the mixed linear model (MLM) using software TASSEL 3.0.124 (http://www.maizegenetics.net/tassel). The population structure was estimated using STRUCTURE 2.3.4 software [49] as in Ren et al. [43]. The pair-wise kinship coefficients were estimated according to the method of Lynch and Ritland [50], performed in the program SPAGeDi [51] (http://ebe.ulb.ac.be/ebe/SPAGeDi.html). The number of permutation runs was set as 10,000 to obtain the permutation-based significance in GLM analysis. MLM was fitted for each marker and phenotype, accounting for Q-Matrix of the population structure as a covariate and pair-wise kinship coefficients (K matrix) as random effects [34]. Significance of associations between marker loci and traits was tested at a corresponding level of the experiment-wise P-value. Significance of associations between loci and traits was described as P-value and the QTL effects were evaluated by marker-R^2^ [52].

## Results

### SNP markers and population structure

Multiplexed 1,536 Illumina Golden Gate SNP assay involving in 150 durum wheat accessions generated 230,400 data points. Out of the examined SNPs, 1,366 (89%) were successfully amplified, and other 10% were missing. The detailed analyses on the SNP markers were reported in Ren et al. [43]. The SNP loci were well distributed across the seven homoeologous chromosome groups. The total marker number ranged from 161 in group 5 to 236 in group 7 chromosomes. The number of polymorphic markers ranged from 108 in group 5 to 161 loci in group 6 chromosomes [43].

The structure analysis was performed in Ren et al. [43], and the result suggested that the observed durum wheat germplasm can be divided into two genetically distinct groups (Group I and Group II). The cluster analysis showed that the group II can be further divided into four subgroups, IIa, IIb, IIc, and IId. The dendrogram of 150 durum wheat landraces based on the shared-allele genetic distance calculated from 1,366 SNP markers was showed in Ren et al. [43].

### Linkage disequilibrium among intra-chromosome SNP loci

A total of 1,338 SNP markers with a mean marker density of 95–96 markers per chromosome, ranging from 66 (3B) to 130 (7A) for all the 14 chromosomes, were used to calculated the extent of LD. The pattern of LD was measured using R^2^ of allele pairs between 2 loci according to Weir and Cockerham [53] on both chromosome and genome levels (Tables 1 and 2).

**Table 1:** SNP locus pairs on the same linkage group with significant (P<0.01) and highly significant (p<0.001) linkage disequilibrium (LD) and R^2^ values at levels of chromosome and genome in durum wheat.

				Locus pairs with significant LD ^a^				
Chromosome	No. of loci	Possible pairs	Observed pairs	P<0.01	P<0.001	R^2^>0.1, P<0.01	R^2^>0.1, P<0.001	Mean R^2^ (%)
**1A**	107	5671	2415	344 (6.07)	213 (3.76)	197 (3.47)	172 (3.03)	3.8
**1B**	97	4656	2775	514 (11.03)	341 (7.32)	292 (6.27)	271 (5.82)	4.1
**2A**	90	4005	2016	455 (11.36)	348 (8.69)	337 (8.41)	323 (8.07)	6.5
**2B**	82	3221	1711	348 (10.80)	244 (7.58)	212 (6.58)	198 (6.15)	5.6
**3A**	98	4753	2145	384 (8.08)	229 (4.82)	262 (5.51)	198 (4.17)	6.4
**3B**	66	2145	1176	225 (10.48)	164 (7.65)	142 (6.62)	136 (6.34)	4.9
**4A**	119	7021	3321	651 (9.27)	451 (6.42)	525 (7.48)	422 (6.01)	8.1
**4B**	72	2556	903	172 (6.73)	117 (4.58)	113 (4.42)	97 (3.80)	5.7
**5A**	83	3043	1653	271 (8.91)	206 (6.77)	195 (6.41)	183 (6.01)	5.3
**5B**	71	2485	946	208 (8.37)	158 (6.36)	146 (5.88)	134 (5.39)	5.9
**6A**	122	7381	2701	463 (6.27)	309 (4.19)	299 (4.05)	251 (3.40)	5.8
**6B**	103	5253	3240	673 (12.81)	421 (8.01)	392 (7.46)	346 (6.59)	6.4
**7A**	130	8385	3655	798 (9.52)	601 (7.17)	575 (6.86)	550 (6.56)	6.9
**7B**	98	4753	2211	401 (8.44)	264 (5.55)	269 (5.66)	232 (4.88)	6.4
**A genome**	749	40259	17906	3366 (8.36)	2357 (5.86)	2390 (5.94)	2099 (5.21)	6.2
**B genome**	589	25069	12962	2541 (10.13)	1709 (6.82)	1566 (6.25)	1414 (5.64)	5.6
**Whole**	1338	65328	30868	5907 (9.04)	4066 (6.22)	3956 (6.06)	3513 (5.38)	6.0

^a^ Number of locus pairs and percentage of all possible locus pairs showing significant LD at P<0.01, P<0.001, R^2^>0.1 & P<0.01, and R^2^>0.1 & P<0.001, respectively.

**Table 2:** SNP locus pairs in different linkage stage with significant (P<0.01) and highly significant (p<0.001) linkage disequilibrium (LD) and R^2^ values on genome level in durum wheat.

					Locus pairs with significant LD				
Genome	No. of loci	Linkage stage	Possible pairs	Observed pairs	P<0.01 (%) ^c^	P<0.001 (%) ^d^	R^2^>0.1 (%) ^e^	R^2^>0.1 (%) ^f^	Mean R^2^ (%)
**A**	749	Linked ^a^	40259	17906	3366 (8.36)	2357 (5.86)	2390 (5.94)	2099 (5.21)	6.2
**A**	749	Unlinked ^b^	239867	106844	18170 (7.58)	12036 (5.01)	11900 (4.96)	10370 (4.32)	5.3
**A**	749	Total	280126	124750	21536 (7.69)	14393 (5.14)	14290 (5.10)	12469 (4.45)	5.4
**B**	589	Linked ^a^	25069	12962	2541 (10.12)	1709 (6.82)	1566 (6.25)	1414 (5.64)	5.6
**B**	589	Unlinked ^b^	148097	74191	13233 (8.94)	8483 (5.73)	7862 (5.31)	7030 (4.75)	4.8
**B**	589	Total	173166	87153	15774 (9.11)	10192 (5.87)	9428 (5.44)	8444 (4.88)	4.9
**Whole**	1338	Linked ^a^	65328	30868	5907 (9.04)	4066 (6.22)	3956 (6.06)	3513 (5.38)	6.0
**Whole**	1338	Unlinked ^b^	829125	390035	67996 (8.20)	44488 (5.37)	42779 (5.16)	37669 (4.54)	5.0
**Whole**	1338	Total	894453	420903	73903 (8.26)	48554 (5.43)	46735 (5.22)	41182 (4.60)	5.1

^a^ Locus on the same linkage group.

^b^ Locus from different linkage groups.

^c^ Locus pairs and percentage of all possible locus pairs showing significant LD at P<0.01.

^d^ Locus pairs and percentage of all possible locus pairs showing significant LD at P<0.001.

^e^ Locus pairs and percentage of all possible locus pairs showing significant LD at R^2^>0.1; P<0.01 adjusted for locus pairs description.

^f^ Locus pairs and percentage of all possible locus pairs showing significant LD at R^2^>0.1; P<0.001 adjusted for locus pairs description.

There were 894,453 possible pair-wise loci in the matrix of 150 genotypes and 1,338 SNP markers. Of these locus-pairs, 5.43% showed significant LD (p<0.001) (Table 2). There were 2,145 (3B) to 8,385 (7A) possible locus pairs in the 14 chromosomes. The percentage of locus pairs showing significant LD (p<0.001) ranged from 3.76% (1A) to 8.01% (6B), respectively. The average R^2^ values varied from 0.038 (1A) to 0.081 (4A) among the 14 chromosomes (Table 1). A small percentage of significant locus pairs had R^2^ value >0.1 (p<0.001). On the average, the highly significant pairs (R^2^>0.1; p<0.001) were 251 per chromosome, ranging from 97 (4B) to 550 (7A). The percentage of all possible locus pairs showing highly significant LD (R^2^>0.1; p<0.001) ranged from 3.03% (1A) to 6.59% (6B) (Table 1). The extent of LD was varying with chromosomes.

Table 2 showed LD value versus genetic distance in the locus pairs on genome level. There were 749 and 589 loci available for LD evaluations in the A and B genome, respectively. Across all 1,338 loci, 65,328 possible pairs of linked loci (in the same linkage groups) and 829,125 pairs of unlinked loci (from different linkage groups) were detected. The observed locus pairs of linked and unlinked loci were 30,868 and 390,935, respectively. Among the linked locus pairs, 2,357 (5.86%) possessed significant LD (P<0.001) in genome A, whereas, 1,709 (6.82%) had significant LD in genome B. As to the unlinked locus pairs, 1,236 (5.01%) had significant LD (p<0.001) in genome A, whereas 8,483 (5.73%) in the B genome.

The mean R^2^ values for all the linked pairs in genome A and B were 0.062 and 0.056, respectively. Therefore, the number of possible pairs, number of significant pairs, and mean R^2^ of the genome A were larger than the genome B except for the percentage of significant pairs (Table 2). The extent of LD was varying with chromosomes. The percentage of significant LD (R^2^>0.1; p<0.001) pairs in the A chromosomes generally was higher than the corresponding B chromosomes except for 1A *vs*. 1B and 6A *vs*. 6B. The mean R^2^ value of the A chromosomes was higher than corresponding B chromosomes except for 1A *vs*. 1B; 5A *vs*. 5B and 6A *vs*. 6B (Tables 1 and 2). Thus the extent of LD of A genome was larger than the B genome on both the chromosome and genome levels in general.

### Variation of the key traits

#### Features of the examined traits

**Fig 1:**
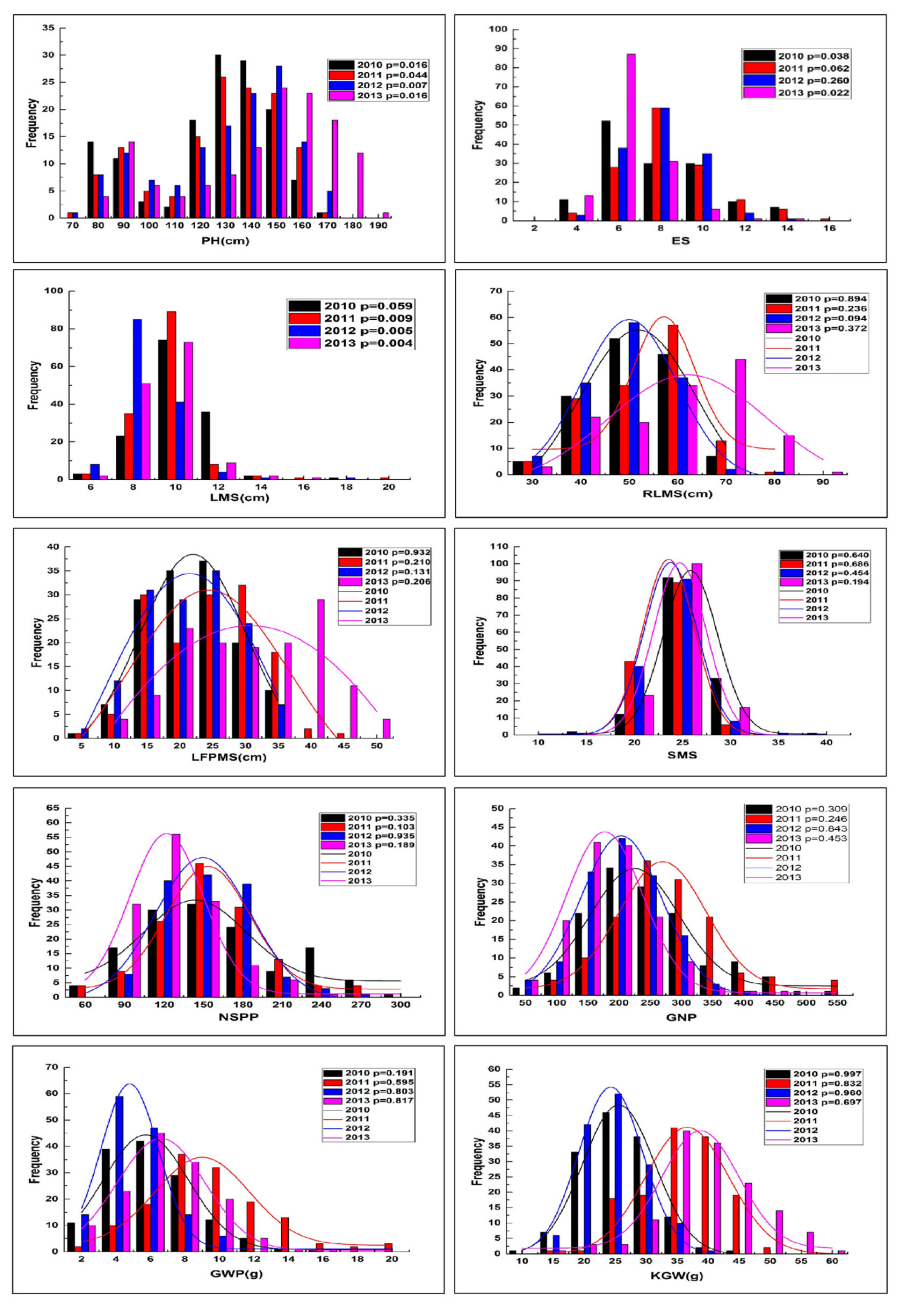
Frequency distribution of the 10 examined agronomic traits of durum wheat in four consecutive years. P value of Kolmogorov-Smirnov test for each year was shown, the hypothesis of normal distribution could be accepted when P>0.05 (significant at P = 0.05), and the trend lines of the accepted normal distribution were shown. PH, plant height (cm); ES, number of effective spikes, LMS, length of main spike (cm); RLMS, rachis internode length of main spike (cm); LFPMS, panicle neck length of main spike (cm); SMS, number of spikelets on main spike; NSPP, number of spikelets per plant; GNP, grain number per plant; GWP, grain weight per plant (g); KGW, 1000-grain weight (g).

All the durum accessions were observed for 10 agronomic and morphological traits in replicated field trials for four consecutive years (Table 3). Distribution histograms of the 10 traits were showed in Fig 1. In general, Kolmogorov-Smirnov test showed that most of the observed traits fitted the normal distribution except for PH, ES and LMS. PH significantly deviated from the normal distribution (P<0.05 in all the 4 years) and showed the feature of binomial distribution. ES significantly deviated from the normal distribution in 2010 and 2013 (P<0.05), and nearly significant in 2011 (P = 0.062). LMS showed significant deviation (P<0.05) in 2011–2013 and nearly significant deviation in 2010 (P = 0.059) (Fig 1). Therefore, most of the observed traits are quantitatively inherited. But PH seems controlled by a single gene together with polygene of minor effects in the population, and distribution of ES and LMS seems varying with the environment.

**Table 3:** Mean values and variation of the 10 examined traits in four consecutive years.

		Trait ^a^									
Year	Item	PH	ES	LMS	RLMS	LFPMS	SMS	NSPP	GNP	GWP	KGW
**2010**	**Average**	121.68	6.88	9.25	46.36	19.46	22.97	144.99	223.21	5.28	23.30
	**CV (%)**	19.19	37.47	19.64	19.44	34.89	13.07	36.31	44.42	51.22	24.35
	**Range**	70–160.25	2.25–13.75	5.75–21.00	21–64.88	-3.50–33.50	14–36.00	55–273	21–562.75	0.2614.74	7.5–41.31
**2011**	**Average**	123.21	7.60	8.64	48.24	21.66	21.09	145.03	257.00	8.52	33.05
	**CV (%)**	19.56	33.29	17.24	21.10	35.68	11.89	33.46	37.45	40.88	19.82
	**Range**	63–160.83	2–22.67	4.88–18.15	24.60–71.00	3.10–41.33	14–28.50	42–386.00	56–650.33	0.83–19.48	13.75–47.85
**2012**	**Average**	125.00	7.08	7.77	44.26	18.93	21.17	136.97	179.39	4.18	22.40
	**CV (%)**	20.72	23.21	17.65	20.57	35.51	11.83	24.31	36.24	44.64	20.83
	**Range**	65.36–167.48	3.33–12.50	4.96–16.55	21.53–71.48	4.37–33.03	15.17–30.17	63.78–253.33	38.33–365.42	0.49–9.53	12.62–38.35
**2013**	**Average**	137.18	5.41	8.39	55.23	27.79	22.23	114.86	159.36	5.84	36.84
	**CV (%)**	21.90	26.55	21.05	23.34	35.97	11.43	27.52	41.54	43.68	21.19
	**Range**	61–183.58	3–12	5.65–22.13	25.25–81.1	5.75–49.4	16.5–29.33	61.5–228.5	13.75–404.75	0.44–14.67	10.19–55.42

^a^ PH, plant height (cm); ES, number of effective spikes, LMS, length of main spike (cm); RLMS, rachis internode length of main spike (cm); LFPMS, panicle neck length of main spike (cm); SMS, spikelets on main spike; NSPP, number of spikelets per plant; GNP, Grain number per plant; GWP, grain weight per plant (g); KGW, 1000-grain weight (g).

#### Trait variation with year and genotype

The trait distribution pattern was similar over the four years, and most of the traits generally showed normal distribution. The year effect was highly significant for most of the observed traits as revealed by the analysis of variance (ANOVA). The genotypic variation was highly significant for all the 10 traits. The genotype × year (G × E) interaction effect was also highly significant for all the examined traits. Estimation of broad-sense heritability (*H* ^*2*^) showed that most of the traits (6/10) have high heritability (*H* ^*2*^>65%) (Table 4). Therefore it is meaningful to conduct association analyses between the traits and SNP markers.

**Table 4:** Analysis of variance and heritability (*H* ^*2*^) of the 10 examined traits.

Trait	Source of variation ^a^	d.f.	MS	F	*H* ^*2*^ (%)
**PH**	**Y**	3	20205.29	411.19**	97.2
	**G**	141	7407.96	150.76**	
	Y * G	423	207.18	4.22**	
	**E**	1135	49.14		
	**T**	1703	16682.25		
**ES**	**Y**	3	358.6	83.87**	57.4
	**G**	141	23.98	5.61**	
	Y * G	423	10.21	2.39**	
	**E**	1135	4.28		
	**T**	1703	53.29		
**LMS**	**Y**	3	155.78	217.61**	91.7
	**G**	141	24.16	33.74**	
	Y * G	423	2	2.80**	
	**E**	1135	0.72		
	**T**	1703	75.36		
**RLMS**	**Y**	3	9334.72	419.17**	93.4
	**G**	141	1083.85	48.67**	
	Y * G	423	72.07	3.24**	
	**E**	1135	22.27		
	**T**	1703	2469.44		
**LFPMS**	**Y**	3	6863.44	396.37**	88.5
	**G**	141	568.42	32.83**	
	Y * G	423	65.22	3.77**	
	**E**	1135	17.32		
	**T**	1703	561.28		
**SMS**	**Y**	3	315.56	124.38**	87.9
	**G**	141	60.42	23.81**	
	Y * G	423	7.33	2.89**	
	**E**	1135	2.54		
	**T**	1703	485.34		
**NSPP**	**Y**	3	81443.16	45.01**	48.5
	**G**	141	8502.39	4.70**	
	Y * G	423	4377.13	2.42**	
	**E**	1135	1809.57		
	**T**	1703	21317.87		
**GNP**	**Y**	3	749025.54	94.43**	56.6
	**G**	141	36798.2	4.64**	
	Y * G	423	15976.96	2.01**	
	**E**	1135	7932.05		
	**T**	1703	55665.68		
**GWP**	**Y**	3	1423.18	138.19**	56
	**G**	141	38.15	3.70**	
	Y * G	423	16.78	1.63**	
	**E**	1135	10.3		
	**T**	1703	52.12		
**KGW**	**Y**	3	19924.11	664.88**	66.4
	**G**	141	235.22	7.85**	
	Y * G	423	79.04	2.64**	
	**E**	1135	29.97		
	**T**	1703	908.17		

** significant at the probability level of 0.01.

^a^ Y, years; G, genotype; E, error; T, total; d.f., degrees of freedom; MS, mean square; *H* ^*2*^: broad-sense heritability.

#### Correlation among the observed traits

Table 5 showed correlation coefficients among the 10 observed traits. Out of the 45 possible correlation pairs, more than 75% (34) were significant or highly significant. LMS, RLMS, LFPMS and SMS showed highly significant positive correlations with PH. NSPP, GNP and GWP showed highly significant positive correlations with ES, while SMS and KGW showed significant and highly significant negative correlations with ES. LMS showed significant positive correlations with RLMS and NSPP. The correlations between LFPMS and GNP, GWP were positive and highly significant. SMS was highly and positively correlated with NSPP, while negatively correlated with KGW. This indicated that the more SMS, the more NSPP correspondingly. In another word, the growth condition of main spike reflected the growth condition of the other spikes to some extent. And the more SMS and NSPP mean lighter and smaller grains. As a result, KGW was negatively correlated with SMS (Table 5).

**Table 5:** Correlation coefficients among the 10 observed agronomic traits.

Trait	PH	ES	LMS	RLMS	LFPMS	SMS	NSPP	GNP	GWP	KGW
**PH**	1									
**ES**	0.056	1								
**LMS**	0.242**	0.195**	1							
**RLMS**	0.848**	-0.015	0.133**	1						
**LFLMS**	0.674**	-0.012	-0.012	0.924**	1					
**SMS**	0.286**	-0.114*	0.326**	0.184**	0.044	1				
**NSPP**	0.169**	0.911**	0.290**	0.073	0.029	0.238**	1			
**GNP**	0.082	0.749**	0.219**	0.112*	0.125**	0.085	0.774**	1		
**GWP**	0.140**	0.534**	0.162**	0.272**	0.300**	-0.005	0.526**	0.832**	1	
**KGW**	0.156**	-0.145**	0.028	0.364**	0.407**	-0.095*	-0.167**	0.026	0.519**	1

*, ** significant at the probability level of 0.05 and 0.01, respectively.

### Association analysis

Association analyses between SNP markers and the 10 quantitative traits (PH, ES, LMS, SMS, RLMS, LFPMS, NSPP, GNP, GWP, and KGW) were conducted preliminarily under the GLM and MLM models by using the computer software TASSEL 3.0.124. Comparison between these two models showed that MLM decreased the total number of significant associations (p<0.01) (data not shown), and most of the significant associations were consistent between the two models. Yu and Buckler [34] suggested incorporating the pair-wise kinship (K matrix) as random effects into a mixed model to correct relatedness and reduce the number of false positives in association analysis. In addition, association analyses in Yang et al [38] and Zhu and Yu [54] indicated that MLM (K+Q) model was better for correcting false positives associations than GLM. Therefore, the results under the MLM model that accounted for both Q and K matrixes were presented in this paper.

**Fig 2:**
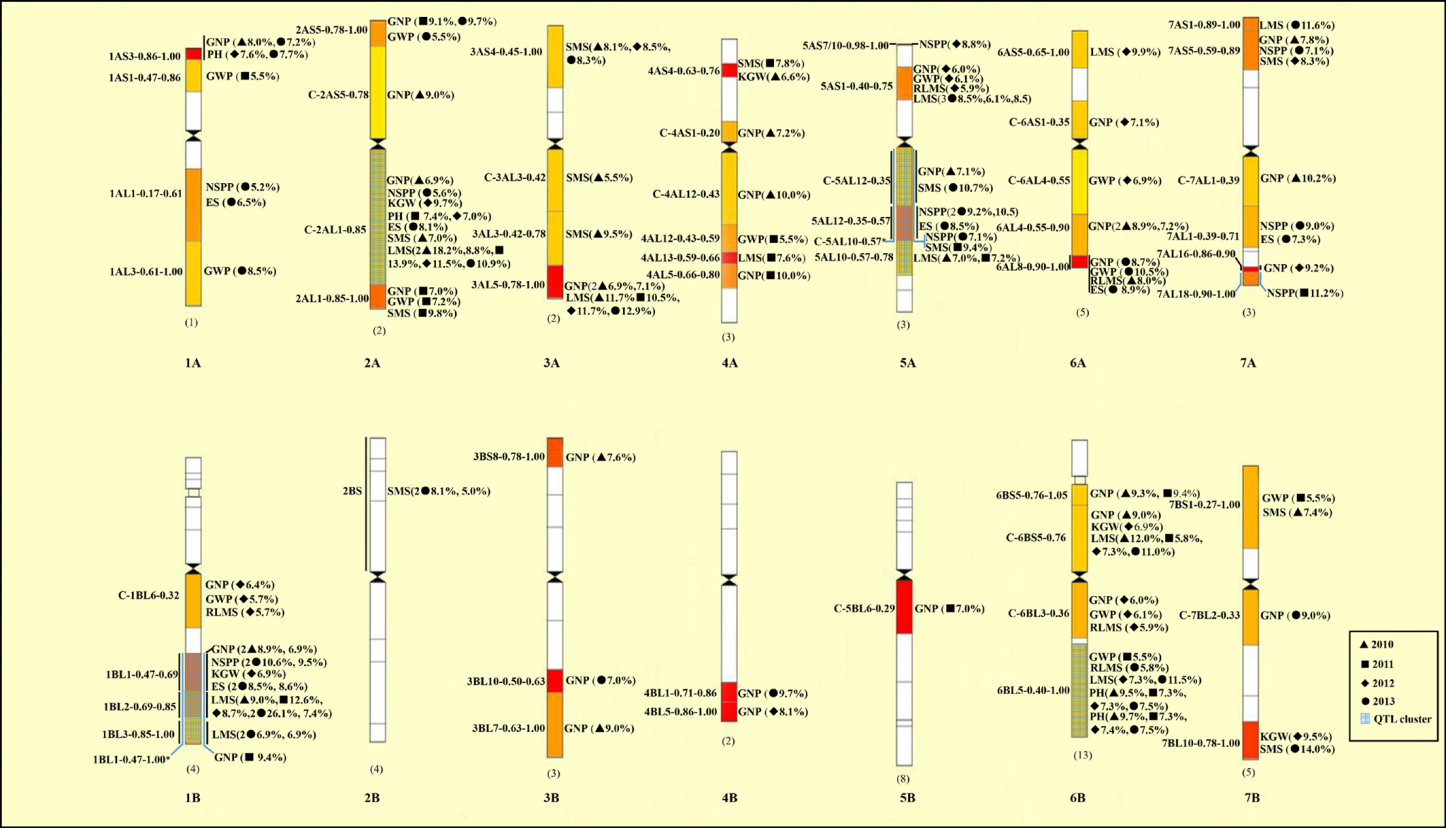
Chromosome bin map of plausible QTLs anchored by SNP markers in durum wheat. The relative interval length is indicated on the left of each chromosome and QTLs represented by SNP-based associations and relative R value (%) are shown on the right. The number in front of the symbol means the repeats of the associations anchored in the interval in the corresponding years and without a number in front of the symbol means one repeat of the association anchored in the interval in one corresponding year. Details of the associations are presented in S2 Table. The exact bins of some associated EST markers are unknown, and thus are shown below the chromosome.

Some imperfect markers were excluded out of the 1,536 SNP markers. Thus 1,366 SNPs were used for association analysis in this study. Table 6 and S2 Table showed an overview and details of trait-marker associations under MLM model in four consecutive years, respectively. Fig 2 is the chromosome bin map showing candidate QTLs anchored by the associated SNP markers in durum wheat. In total, 201 significant associations were detected in the four years (60, 26, 45 and 70 for the year 2010, 2011, 2012 and 2013, respectively). The associations between SNP markers and traits were varying with the years.

**Table 6:** Number of associated SNP markers in different years for the examined traits.

	Number of associated SNP markers				
Trait	2010	2011	2012	2013	Total
**PH**	4	4	6	5	19
**ES**	0	1	0	12	13
**LMS**	10	6	9	15	40
**RLMS**	1	0	3	1	5
**LFPMS**	1	0	0	0	1
**SMS**	8	3	4	9	24
**NSPP**	0	1	2	13	16
**GNP**	33	5	10	10	58
**GWP**	0	6	7	5	18
**KGW**	3	0	4	0	7
**Total**	60	26	45	70	**201**

In 2010, sixty markers were significantly associated with the ten observed traits. The distributions of the association pairs were uneven among the traits. Most of the associations were detected between markers and the yield traits. More than half of the markers were associated with GNP, and the number of associated markers for other traits range from 1 (ES, LFPMS and RLMS) to 10 (LMS). The percentage of the variation explained by marker ranged from 5.4% (*CD454448_6_A_84* associated with KGW) to 18.2% (*BG605368_2_A_Y_310* associated with LMS).

In 2011, we detected 26 marker-trait association pairs. The number of the associated markers ranged from 1 (ES and NSPP) to 6 (LMS and GWP) (Table 6). The percentage of the variation explained by marker was in a range between 5.4% (*BG274294_1_B_382* associated with SMS) and 13.1% (*BG605368_2_A_Y_310* associated with LMS).

In 2012, 45 marker-trait associations were detected. The number of the associated markers ranged from 2 (NSPP) to 10 (GNP). The percentage of the variation explained by marker varied from 5.2% (*BG312827_6_A_Y_305* associated with PH) to 11.6% (*BM134437_3_A_Y_233* associated with LMS).

For the year 2013, 70 associations were detected. The percentage of the total variation explained by marker varied from 5.0% (*BE444144_2_B_N_138* associated with SMS) to 26.1% (*BF474284_1_B_Y_357* associated with LMS) (Table 6, S2 Table).

Moreover, taking consideration of all the four years, the number of markers associated with each trait ranged from 1 (LFPMS) to 54 (GNP), and the percentage of the total variation explained by marker ranged from 5.0% (*BE444144_2_B_N_138* associated with SMS) to 26.1% (*BF474284_1_B_Y_357* associated with LMS) (S2 Table). We found that one trait associated with many markers (e.g., GNP with 54 markers), and single markers were associated with multiple traits (*BE590553_7_A_190* associated with GNP, NSPP and SMS, and *BE443538_5_A_1436*, *BE590521_6_B_N_331* associated with GNP, GWP and RLMS, etc.). This may indicate that quantitative traits are always conferred by multiple loci, and QTLs conferring multiple agronomic traits may cluster around the single regions/markers due to pleiotropic effects of genes [55]. Seven associations (4 for LMS, 3 for PH) were detected in all the four years. Two associations (1 for PH, and 1 for SMS) were detected in three of the four years. Eleven associations were detected in two of the four years (S2 Table). These reproducible associations were significant and more reliable.

#### Associations for morphological traits

Plant height (PH): six significantly associated SNPs were detected in four years of 2010–2013 (Table 6). Three SNP markers, *BE405269_4_B_84*, *BF475120_6_B_67*, and *BF475120_6_B_Y_75* were detected to be significantly associated with PH in all the four years. Other three SNPs, *BG312827_6_A_Y_305*, *BE443948_2_A_Y_345* and *BE490041_1_A_371* were significantly associated with PH in three or two of the four years (S2 Table). Furthermore, PH showed feature of the binomial distribution (Fig 1) and thus may be controlled by the polygene including a single major gene and some minor genes in the populations. These PH-associated SNP markers were mainly located in chromosome 1A, 2A, 4B, 6A and 6B. Several marker loci, significantly associated with PH, were previously detected on chromosomes 4B, 5A, 5B, 6B, 7A and 7B [56].

RLMS and LFPMS: A total of 5 and 1 SNP markers were detected in the four years for RLMS and LFPMS, respectively (Table 6, S2 Table). Markers significantly associated with the traits were present on chromosome 1B, 5A, 6A and 6B. *BE443538_5_A_1436*, *BE590521_6_B_N_331* and *BG314205_1_B_33* were associated with RLMS, GNP and GWP. Correlation analysis indicated significant positive correlations of RLMS with GNP and GWP (Table 5). Flag leaf and rachis internode were related to photosynthesis and photosynthetic product accumulation and transfer, and thus played important roles in grain filling process [57]. Therefore, it is understandable that SNP markers associated with RLMS and LFPMS are also related with GNP and GWP.

LMS: Six to fifteen associations were detected between LMS and SNP markers in the four years (Table 6, S2 Table). The SNP markers associated with LMS were located on chromosome 1B, 2A, 3A, 4A 5A, 6A, 7A and 6B. Four SNP markers *BE445667_6_B_Y_285*, *BF474284_1_B_Y_357*, *BG605368_2_A_Y_310* and *BM134437_3_A_Y_233*, were significantly associated with LMS in all the four years. Five SNPs showed significant associations with LMS in two of the four years (S2 Table). The marker *BF484028_5_A_Y_97* corresponding to the *Vrn-A1* region in the interval of 5AL10-0.57–0.78 was significantly associated with LMS. Some associations were founded to be located in the same regions for LMS-related traits (GNP and GWP etc.) (Table 5, Fig 2).

#### Associations for yield traits

ES and NSPP: A total of 13 and 16 SNP markers were associated with ES and NSPP in the four years, respectively (Table 6, S2 Table). Some SNP markers were associated with both ES and NSPP. Highly significant positive correlation was detected between ES and NSPP (Table 5).

SMS, GNP and GWP: A total of 22, 54 and 18 significant associations with SNP markers were detected for SMS, GNP and GWP in the four years, respectively (Table 6, S2 Table). *BG314551_3_A_Y_162* was significantly associated with SMS in three of the four years. This SNP explained over 8.1% of the variation (Table 6, S2 Table). The EST represented by *BG314551_3_A_Y_162* was located in the same region as *Eps* gene (earliness per se). GWP showed positive correlation with GNP. Several SNP markers are thus associated with both GNP and GWP.

KGW: A total of 7 significant associations between KGW and SNP markers were detected in all the four years. These SNP markers associated with KGW were located in chromosomes 1B, 2A, 4A, 5B, 6A, 6B and 7B (R^2^ = 4.9–9.7%), and mainly located in chromosomes 2A, 5B, 6A, 7A and 7B with R^2^>9.2% (S2 Table). Peng et al. [55] found eight QTLs for GWH (100-grain weight) on chromosomes 1B, 2A, 4A, 5A, 5B, 6B, 7A, and 7B, and major GWH QTLs were located on chromosomes 2A, 4A, and 5B. The marker *AY244508_5_B_Y_26*, significantly associated with KGW and GNP, was located in the same region as *AP1* and *Vrn-B1*.

## Discussion

### Linkage disequilibrium in durum wheat

The variation patterns of LD at both the chromosome and genome levels reflect the complicated evolutionary and breeding history in wheat [58]. In the present study, we demonstrated an extensive amount of LD in durum wheat using 1,338 SNP markers (Tables 1 and 2).

The extent of LD in A genome is higher than in B genome in general. The similar result was reported in previous study [59]. In their study based on SSR markers, the highest extent of significant LD was observed in D genome, followed by the A and B genomes of the bread wheat [59].

The genomic locations of genes controlling important adaptive traits were different. These can have a differential influence on LD in different genomes. *Vrn-A1* gene on chromosome 5A has higher number of widely distributed haplotypes than the *Vrn-B1* gene on chromosome 5B and thus more likely to have a stronger effect on LD [60]. In our study, chromosome 4B had the lowest percentage of significant LD pairs and mean R^2^ value, and thus possessed relatively low LD extent in chromosome 4B (Table 1). Akhunov et al. [61] also reported that chromosome 4B had the lowest number of haplotypes per locus and lowest haplotype diversity. This may indicate that the haplotype diversity and genes controlling important adaptive traits have a differential influence on LD in chromosome 4B. Therefore, the divergence in the extent of LD is probably related to breeding history and selection pressure applied to genes located in the different chromosomes and genomes during the process of cultivation [62].

The genetic diversity of genome A is lower than genome B [43, 55]. The extant LD in genome A is higher than in genome B, on the contrary. On chromosome level, some chromosomes have the similar extant LD (like 2A and 2B, 3A and 3B, 4A and 4B etc.) (Table 1). Chao et al [62] reported similar result. The extant LD was related to genetic diversity in the individual breeding program. The domestication history of genome A is longer than genome B in wheat [55, 63]. Genome A thus probably has more genes controlling important adaptive traits. Under the natural and artificial selections in the breeding programs, the genome A of cultivars captured comparable number of adaptive traits/genes, and widely distributed haplotypes resulting from the high extant LD [62, 63]. As mentioned above, breeding/domestication history and selection specific to each breeding program have influence on LD to some extent.

### Candidate QTLs revealed by association analysis

In the present study we performed association analysis using big number of SNP markers in durum wheat consisting of worldwide accessions. A total of 201 association pairs between SNP markers and 10 quantitative traits were detected in the four years (S2 Table). Fifty-two known regions were marked on the 14 chromosomes (Fig 2), which may represent the candidate QTLs.

Four credible SNP associations for PH were reproducible at least in three of the four consecutive years. These associations were located on 4B, 6A and 6B. Two markers (*BF475120_6_B_67* and *BF475120_6_B_Y_75*) located on the same position in the region 6BL5-0.40–1.00 of the long arm of chromosome 6B, were associated with PH in all of the four years, and these two associations possibly represent a single credible QTL explaining over 7.2% of the variation in the four years (S2 Table). Several QTLs were reported in the similar region of 6BL by Börner et al. [64] and Cadalen et al. [56].

Four credible associations for LMS were reproducible in the four consecutive years. These associations were located on 1B, 2A, 3A and 6B, respectively, and thus might represent 4 QTLs. *BG605368_2_A_Y_310*, located on 2AL, was associated with LMS and explained 10.8% of the variation in the four years (S2 Table). Similar QTL for LMS was detected in the region of 2AL using SSR and EST-SSR markers in Yao et al [52], and Peng et al. [55] mapped over ten QTLs involving similar traits (PH, GNP, KGW and LMS) and defined two domestication factors in this chromosome arm. *BE445667_6_B_Y_285*, located on 6BL, was associated with LMS in the four years (S2 Table). QTLs involving similar traits (PH, GNP, KGW and LMS) were detected also in this region by Börner et al. [64].

The credible candidate QTLs may reside in a region containing several candidate genes conferring the examined traits. The candidate genes may have pleiotropic effects or several genes are clustered in the same region and acting on different traits [55]. Therefore, the candidate QTLs or the QTL-carried regions are potential reference regions for gene cluster. These QTLs and the clustering regions are worthy of further precisely QTL locating and gene detecting and cloning.

### QTL clusters in the genome

As shown in Fig 2, most of the SNP associations were located on chromosomes 2A, 5A, 1B and 6B. The number of association effects in the A genome was larger than that in the B genome (Table 1, Fig 2). The genome A has longer domestication evolution history than the genome B in wheat, and thus probably has more genes controlling important adaptive traits [1, 55]. Chao et al. [62] demonstrated that the genome A of wheat cultivars captured comparable number of adaptive trait genes under the natural and artificial selection and in the breeding programs.

It is noteworthy that several associations co-locate in the same chromosome regions, even for the unrelated traits. There are several regions with association clusters especially on chromosomes 2A, 5A, 6A, 7A, 1B and 6B. For example, seven associations for PH, GNP, KGW and LMS are located on the proximal region C-2AL1-0.85 of chromosome 2 (S2 Table, Fig 2). Peng et al. [55] mapped over ten QTLs involving similar traits (PH, GNP, KGW and LMS) and defined two domestication factors in this chromosome arm. Yao et al. [52] detected similar QTLs for spike length, thousand kernel weight and spike number per plant in the same region. This region may be a convincible region for cluster of QTLs.

On the chromosome 5A, we detected association clusters for LMS, GNP, GWP and SMS mainly in the short arm (5AS1-0.40–0.75) and the long arm (5AL12-0.35–0.78) (Fig 2). Kato et al. [65] and Gadaleta et al. [66] reported QTL clusters for yield components (thousand kernel weight, grain yield per spike and kernel number per spike) in similar region 5AL15-0.67–0.78. Peng et al. [55] mapped 19 QTLs involving 11 traits including LMS, GNP, GWP and SMS and also defined two domestication factors in this chromosome 5AL arm. In Gadaleta et al. [66], many SNPs mapped in the bin 5AS1-0.40–0.75 on the short arm have duplicated loci in bin 5AL5-0.46–0.55 on the long arm. The bin on 5AS may have undergone a duplication followed by an insertion into the 5AL of the same chromosome 5A. This may explain the similar associations mapped in the regions of 5AS1-0.40–0.75 and 5AL12-0.35–0.78 (Fig 2).

Another significant cluster of associations for PH, GNP, KGW and LMS was detected on the long arm of chromosome 1B (1BL1-0.47–1.00) (Fig 2). Similarly, Börner et al. [64] detected QTLs for spike length and grain weight in this region. Similar result was reported by Cadalen et al. [56]. Peng et al. [55] mapped 8 QTLs involving 8 traits including LMS, GNP, GWP and SMS and defined one domestication factor in this 1BL chromosome arm.

Phenomenon of QTL clustering was formally reported by Peng et al. [55] for domestication-related traits in wild emmer wheat. They defined a cluster of QTLs co-located in the same chromosome region as domestication syndrome factor [55]. Actually this phenomenon of QTL clustering was repeatedly observed, although not verbally using the term of ‘QTL cluster’, in wheat [52, 56, 64–68]. In the present study, we demonstrated obvious QTL clusters represented by SNP-based associations in durum wheat (Fig 2). More and more studies tend to show that genes often reside in the genome in clusters. This seems especially true for resistance genes and QTLs for quantitatively inherited traits. The genetic mechanism for this universal phenomenon is the pleiotropic effect of genes [55]. Nevertheless, the genomic regions of QTL clusters need further validation by fine mapping and cloning of QTLs or genes.

### Genes for plant height

Plant height (PH) is the key agronomic trait in wheat. We found six marker-trait associations for PH located on chromosomes 1A, 2A, 4B, 6A and 6B in four years. Each of the two markers, *BF475120_6_B_67* and *BF475120_6_B_Y_75*, associated with pH explained >7.0% of variation in four years (S2 Table). In the chromosome region 6BL5-0.40–1.00 of *BF475120* (http://wheat.pw.usda.gov/GG2/index.shtml), the SSR marker *Xfbb250-6B* was founded to be significantly associated with PH [56]. As shown in NCBI database (http://www.ncbi.nlm.nih.gov/), *BF475120* is an EST sequence fragment derived from wheat salt-stressed crown cDNA library. The encoded protein of *BF475120* has very high homology (E = 1e^-53^) with the protein GDSL esterase/lipase from *Aegilops tauschii*. One member of rice GDSL esterase family might be involved in lipid yield [69]. Esterase/lipase is involved in the entire process of plant growth and development. Furthermore, Börner et al [64] detected two QTLs for PH on the similar region 6BL5-0.40–1.00 of 6BL. Thus it is reasonable that *BF475120* is associated with PH.

The SNP marker *BG312827_6_A_Y_305* associated with PH explained >5.2% of variation in the four consecutive years. The EST *BG312827* was derived from *T*. *monococcum* early reproductive apex cDNA library (http://www.ncbi.nlm.nih.gov/). The encoded protein has very high homology (E = 1e^-63^) with the DNA replication licensing factor, a mcm5-A-like enzyme from *Brachypodium distachyon* (http://www.ncbi.nlm.nih.gov/). DNA replication licensing factor expressed in shoot apex and flower buds is essential to undergo a single round of replication initiation and elongation per cell cycle [70]. *Arabidopsis MCM2* to *MCM5* and *MCM7* genes contain E2F consensus sites in their promoters. Their transcripts are elevated in plants expressing E2FA/DPA which not only regulates the mitotic cell cycle progression but also plays a role in the endocycle. It is a prerequisite for normal plant development [70–72]. Therefore *BG312827* closely relates with apex cell division and growth, and thus undoubtedly associate with PH.

Additionally, the marker *BE405269_4_B_84* without exact site, located on chromosome 4B, was associated with PH in all the four years. This reproducible significant association is reliable. *Rht-B1*, located on chromosomes 4BS, is known to have major effect on PH [73]. The marker *BE405269_4_B_84* was located in the same chromosome with *Rht-B1*, while the exacted region and relations need to be further explored.

### Genes for length of main spike

For length of main spike (LMS), we found a total of 23 SNP associations located on chromosomes 1B, 2A, 3A, 4A 5A, 6A, 7A and 6B in the four years. These reproducible associations are significant and reliable. *BF484028_5_A_Y_97* associated with LMS (S2 Table), and was mapped in the interval of 5AL10-0.57–0.78 (http://wheat.pw.usda.gov/GG2/index.shtml). Two genes *Vrn-A1* and *Fr1*, are located in the same chromosome interval as *BF484028_5_A_Y_97* [74]. *Vrn-A1*, a member of *Vrn-1* genes, regulates flowering-time, an important criterion for regional adaptation and yield in all the cereal crops [75]. *Vrn-1* gene is associated with heading date, spike length and grain yield. *Vrn-A1* had a greater effect on spike length [75–77]. Furthermore, *Vrn-1* completely links to MADS-box gene *AP1* [78] which defines the pattern of where floral organs arise, as well as determines development of the floral meristem [79, 80]. Therefore, the gene marked by *BF484028_5_A_Y_97* may affect LMS through *Vrn-A1* gene regulating vernalization.

The marker *BF474284_1_B_Y_357* associated with LMS explained >8.6% of the variation in the four consecutive years. *BF474284* is an EST derived from wheat vernalized crown cDNA library. It has complete homology (E = 0.0) with *TAVDAC2* gene located on the long arm of chromosome 1B in wheat (http://www.ncbi.nlm.nih.gov/). The *Tavdac* cDNAs express in meristematic tissues (floral tissues and embryos), regulate the mitochondrial functions during the period of floral development to embryo formation [81]. Therefore, *Tavdac* is indirectly related to floral development and embryo formation in some ways, e.g., regulating the mitochondrial functions. This explained why *BF474284* was associated with LMS to some extent.

### Gene for number of spikelets on main spike

For number of spikelets on main spike (SMS), we found a total of 22 significant associations in the four years. One reliable SNP marker *BG314551_3_A_Y_162*, significantly associated with SMS in three years, explained over 8.1% of the variation (S2 Table). This SNP was located in the bin 3AS4-0.45–1.00 on chromosome arm 3AS in the same region as *Eps* gene (earliness per se). This gene is usually responsible for the fine-tuning of wheat flowering time. RFLP markers linked with *Eps* explained significant variation of plant height, thousand kernel weight, kernel number per spike, and grain yield [82, 83]. Thus *BG314551_3_A_Y_162* represent a significant factor from early reproductive apex greatly impacting SMS.

### Candidate gene for grain number per plant

Grain number per plant (GNP) is a key yield component factor in wheat. A total of 54 significant SNP associations were detected for GNP in the four years. Several reliable QTLs could be suggested for this trait (Table 6, S2 Table). *BF293541_4_A_Y_88* is located in the bin 4AL5-0.66–0.80 on chromosome arm 4AL (http://wheat.pw.usda.gov/GG2/index.shtml). This region was associated with spike length, spikelets density, grain number per spike [84].

The EST of *BF202706_4_A_Y_466* derived from wheat pre-anthesis spike cDNA library was mapped to wheat deletion bin 4AL12-0.43 (http://wheat.pw.usda.gov/GG2/index.shtml). This region harbors QTLs for grain yield, grain filling rate, spike length and grain number/m^2^ [64, 85].

The EST of *BE498418_7_A_148* was also derived from pre-anthesis spike cDNA library and mapped on 7AL (C-7AL1-0.39). This EST has very high homology (E = 1e^-104^) with UDP-D-xylose epimerase 3 coded by *UXE3* gene from *UXE* gene family in *Hordeum vulgare* (http://www.ncbi.nlm.nih.gov/). The abundant transcript of *HvUXE* was possibly correlated to arabinoxylan deposition in cell walls in the starchy endosperm during grain development. There was a substantial increase in *HvUXE1* and *HvUXE3* mRNA levels at the differentiation stage of endosperm development [86, 87]. The chromosome region of *BE498418* was also proved to carry the QTL for grain weight [64]. This further confirms the association of *BE498418_7_A_148* with GNP.

The EST of *BG263521_2_A_61* mapped in chromosome bin C-2AS5-0.78 (http://wheat.pw.usda.gov/GG2/index.shtml), was also derived from wheat pre-anthesis spike cDNA library, and has very high homology (E = 2e^-126^) with putative serine/threonine-protein kinase *WNK1* (http://www.ncbi.nlm.nih.gov/). *WNK1* gene is member of *WNK* gene family, which involved in the regulation of flowering time in *Arabidopsis* [88]. Several QTLs for grain yield and kernel number per spike were detected within this region [89]. Therefore, the associations between *BG263521_2_A_61* and GNP may be true. Gene marked by SNP *BG263521_2_A_61* affects GNP by regulating flowering time just as *WNK* does.

### Candidate gene for the 1000-grain weight

The 1000-grain weight (KGW) is another key yield component factor. A total of 7 significant associations between KGW and SNP markers mainly located in chromosomes 2A, 5B, 6A, 7A and 7B with R^2^>5.4%, were detected in all the four consecutive years (S2 Table). The SNP marker *AY244508_5_B_Y_26*, significantly associated with KGW and GNP and explained over 11% of variation, was located in the same region as *AP1* and *Vrn-B1*. *AP1* defines the genesis pattern of floral organs, as well as determines development of the floral meristem [79, 80]. *WAP1*, a wheat *APETALA1* homolog, plays a core role in the phase transition from vegetative to reproductive growth [90, 91]. Therefore, associations of *AY244508_5_B_Y_26* with KGW and GNP may be attributed to the role of *AP1* and *VRN1*.

Furthermore, in the composite map of wheat chromosome 5B (http://wheat.pw.usda.gov/GG2/index.shtml), three QTLs (*QGpc*.*ndsu-5B*.*1*, *QYld*.*ndsu-5B* and *QGw1*.*inra-5B*) lie in the interval *Xmwg922–Xcdo1326*.*1* affect KGW and grain yield around the *Vrn-B1* locus [92, 93]. Thus there may be many loci on chromosome 5B controlling grain weight.

*BG605368_2_A_Y_310* was associated with KGW, and explained 9.71% of variation (S2 Table). As discussed above, *BG605368_2_A_Y_310* was also associated with LMS in all the four years. The EST *BG605368* was derived from wheat pre-anthesis spike cDNA library. It is highly homologous (E = 1e^-127^) with Exopolygalacturonase from *T*. *urartu*. Exopolygalacturonase expressed in pollen and young developing tissues, suggesting that they could be implicated in the cell wall modifications and related to cell elongation and/or expansion in these tissues [94]. *BG605368* may be related to flower development. Several QTLs for grain weight and yield in the region (C-2AL1-0.85) of the EST were detected in previous study [64, 95, 96]. Therefore, the association between *BG605368_2_A_Y_310* and KGW and LMS should be credible.

## Conclusions

The previous studies indicated that both QTL analysis and association mapping are suitable and effective tools for mapping quantitative loci in wheat and barley [7, 9, 55, 97–99]. We detected 201 significant associations in total between SNP markers and 10 quantitative traits in durum wheat in four years. Some of the associations are corroborated by the previous QTL analyses, and further supported by the functions of the deriving ESTs and the homologous genes. The plausible QTLs represented by the associated SNP markers are generally clustered in specific chromosome regions of the wheat genome, especially 2A, 5A, 6A, 7A, 1B, and 6B chromosomes. Nevertheless, the associated SNP markers need to be further confirmed before they can be utilized in marker-assisted selection breeding programs [7, 9, 100].

## Supporting Information

S1 Table Durum wheat accessions used in the study. Accession identifier, accession name, place of origin and year of collection are listed for each of the 150 entries.
(DOCX)

S2 Table Significant trait-SNP marker pairs in four consecutive years. ^a^ PH, plant height; ES, number of effective spikes, LMS, length of main spike; RLMS, rachis internode length of main spike; LFPMS, pillow neck length of main spike; SMS, spikelets on main spike; NSPP, number of spikelets per plant; GNP, Grain number per plant; GWP, grain weight per plant; KGW, 1000-grain weight; ^b^ P: the permutation based test for marker significance of individual markers; ^c^ R^2^: the fraction of the total variation explained by the marker after fitting the other model effects.
(DOCX)
