# Supplementary material for: Association of Agronomic Traits with SNP Markers in Durum Wheat (Triticum turgidum L. durum (Desf.))
Source: PLoS One. 2015 Jun 25;10(6):e0130854. doi: 10.1371/journal.pone.0130854 (PMC4482485; doi:10.1371/journal.pone.0130854)
Supplement: S1 Table — Accession identifier, accession name, place of origin and year of collection are listed for each of the 150 entries. (DOCX) [file pone.0130854.s001.docx]

**S1 Table.** **Durum wheat accessions used in the study. Accession identifier, accession name, place of origin and year of collection are listed for each of the 150 entries.**

| **Code#** | **Accession identifier#** | **Accession name** | **Place of origin** | **Year of collection** | |
| --- | --- | --- | --- | --- | --- |
| **H1** | CItr 11495 | Wash. No. 2628 | Heilongjiang, China | 1932 | |
| **H2** | CItr 12068 | Kubanka 314 | North Dakota, United States | 1940 | |
| **H3** | CItr 13246 | Ramsey | North Dakota, United States | 1955 | |
| **H4** | CItr 13333 | Wells | North Dakota, United States | 1957 | |
| **H5** | Ldn 16 |  | North Dakota, United States | 1900 | |
| **H6** | CItr 15874 | D 19329-28M-11Y | Mexico | 1972 | |
| **H7** | CItr 17057 | CAR 1131 | La Araucania, Chile | 1972 | |
| **H8** | CItr 17058 | CAR 1132 | La Araucania, Chile | 1972 | |
| **H9** | CItr 17157 | CAR 1232 | La Araucania, Chile | 1972 | |
| **H10** | CItr 17159 | CAR 1234 | La Araucania, Chile | 1972 | |
| **H11** | CItr 17337 | Wakooma | Saskatchewan, Canada | 1974 | |
| **H12** | CItr 2468 |  | Germany | 1904 | |
| **H13** | CItr 3267 | Chistunka | Altay, Russian Federation | 1911 | |
| **H14** | CItr 5077 | FHB4495 | China | 1916 | |
| **H15** | CItr 5083 | FHB4501 | China | 1916 | |
| **H16** | CItr 5094 | FHB4512 | Beijing, China | 1916 | |
| **H17** | CItr 5136 | Indian Runner | Victoria, Australia | 1916 | |
| **H18** | CItr 6881 | Akrona | Colorado, United States | 1923 | |
| **H19** | CItr 8327 | Suifu | Sichuan, China | 1924 | |
| **H20** | PI 107606 | Cadia | Australia | 1934 | |
| **H21** | PI 109588 | T-538 | Ankara, Turkey | 1935 | |
| **H22** | PI 11715 | Marouani | Mascara, Algeria | 1904 | |
| **H23** | PI 124292 | ICARDA-IG-82575 | Jiangsu, China | 1937 | |
| **H24** | PI 134596 | Fere-Alexandrinum | Syria | 1939 | |
| **H25** | PI 140184 | ICARDA-IG-82637 | Khuzestan, Iran | 1941 | |
| **H26** | PI 152567 | Aden | Yemen | 1945 | |
| **H27** | PI 153774 | Durum H | Giza, Egypt | 1946 | |
| **H28** | PI 165846 | Amarah | Iraq | 1948 | |
| **H29** | PI 168692 | Muestra 2 Barba Blanca Anquipa | Peru | 1948 | |
| **H30** | PI 168708 | Barrigon Glabrous Selection | Mexico | 1948 | |
| **H31** | PI 174645 | Huguenot | Western,Australia, Australia | 1949 | |
| **H32** | PI 176228 | ICARDA-IG-84631 | Nepal | 1949 | |
| **H33** | PI 182697 | Nashabie | Dimashq, Syria | 1949 | |
| **H34** | PI 191645 | Timor | Sao Paulo, Brazil | 1950 | |
| **H35** | PI 192711 | Ostpreuss | Gotland, Sweden | 1950 | |
| **H36** | PI 193391 | Aleppo | Halab, Syria | 1951 | |
| **H37** | PI 208903 | Rash Kool | Iraq | 1953 | |
| **H38** | PI 208907 | Lara | Iraq | 1953 | |
| **H39** | PI 208908 | Mendola | Iraq | 1953 | |
| **H40** | PI 208910 | Sin El-Jamil | Iraq | | 1953 |
| **H41** | PI 210910 | T 1 | Punjab, Pakistan | | 1953 |
| **H42** | PI 210911 | T 2 | Punjab, Pakistan | | 1953 |
| **H43** | PI 210952 | Damliko | Cyprus | | 1953 |
| **H44** | PI 222675 | ICARDA-IG-85523 | East Azerbaijan, Iran | | 1954 |
| **H45** | PI 233213 | Sevindz | Azerbaijan | | 1956 |
| **H46** | PI 235159 | Giza | New South Wales, Australia | | 1956 |
| **H47** | PI 237632 | Tripolitico | Cyprus | |  |
| **H48** | PI 243790 | ICARDA-IG-85615 | Tehran, Iran | | 1957 |
| **H49** | PI 249816 | N-163 | Israel | | 1958 |
| **H50** | PI 249820 | Neveh Yaar 51 | Israel | | 1958 |
| **H51** | PI 253801 | K918 | Ninawa, Iraq | | 1958 |
| **H52** | PI 278223 | Gartons Early Cone | England, United Kingdom | | 1962 |
| **H53** | PI 278648 | ICARDA-IG-85863 | England, United Kingdom | | 1962 |
| **H54** | PI 283853 | China 34 | China | | 1962 |
| **H55** | PI 286546 | Morocho Colorado | Pichincha, Ecuador | | 1963 |
| **H56** | PI 289821 | ICARDA-IG-97583 | Fars, Iran | | 1963 |
| **H57** | PI 292035 |  | Israel | | 1963 |
| **H58** | PI 306664 | Heines Hartveizen | Lower Saxony, Germany | | 1965 |
| **H59** | PI 321702 | Nursi | England, United Kingdom | | 1967 |
| **H60** | PI 344743 | Apulicum 233 | Bulgaria | | 1969 |
| **H61** | PI 345707 | Sevindz | Azerbaijan | | 1969 |
| **H62** | PI 346985 | Hacimestan | Turkey | | 1970 |
| **H63** | PI 352377 | T-357 | Switzerland | | 1969 |
| **H64** | PI 352389 | Caravicos | Greece | | 1969 |
| **H65** | PI 352390 | T-842 | Tunisia | | 1969 |
| **H66** | PI 352395 | T-1303 | Ethiopia | | 1969 |
| **H67** | PI 352404 | Torcal | Spain | | 1969 |
| **H68** | PI 352408 | T-1560 | Italy | | 1969 |
| **H69** | PI 352415 | Aziziah 17/45 | Latium, Italy | | 1969 |
| **H70** | PI 352436 | T-2114 | Former Soviet Union | | 1969 |
| **H71** | PI 352437 | T-2115 | Former Soviet Union | | 1969 |
| **H72** | PI 352450 |  | France | | 1969 |
| **H73** | PI 352551 | Abyssinicum | Ethiopia | | 1969 |
| **H74** | PI 376498 | DF 14/71 | Romania | | 1972 |
| **H75** | PI 376500 | DF 31/71 | Romania | | 1972 |
| **H76** | PI 376501 | DF 42/71 | Romania | | 1972 |
| **H77** | PI 376509 | DF 4/72 | Romania | | 1972 |
| **H78** | PI 376511 | DF 6/72 | Romania | | 1972 |
| **H79** | PI 376512 | DF 7/72 | Romania | | 1972 |
| **H80** | PI 377882 | Duramba | Australia | | 1973 |
| **H81** | PI 384043 | Merarit | Israel | | 1973 |
| **H82** | PI 388035 | Line 76 | Israel | | 1974 |
| **H83** | PI 388132 | FAO 33.268 | Punjab, Pakistan | | 1974 |
| **H84** | PI 41015 | Jalalia | Madhya Pradesh, India | | 1915 |
| **H85** | PI 41342 | Hansia Broach | Gujarat, India | | 1915 |
| **H86** | PI 422289 | Maghrebi 72 | Mexico | | 1978 |
| **H87** | PI 42425 | Zwartbaard | South Africa | | 1916 |
| **H88** | PI 428453 | Dommel‘S’ | Federal District, Mexico | | 1978 |
| **H89** | PI 428701 | AUS 20299 | Australia | | 1978 |
| **H90** | PI 435100 | Bian Sui | China | | 1979 |
| **H91** | PI 438973 | Har'kovskaja 51 | Kharkiv, Ukraine | | 1980 |
| **H92** | PI 447421 | ST-33 | Xinjiang, China | | 1980 |
| **H93** | PI 45442 | ICARDA-IG-98118 | Free State, South Africa | | 1917 |
| **H94** | PI 45443 | ICARDA-IG-98119 | Cape Province, South Africa | | 1917 |
| **H95** | PI 46766 | Golden Ball | Cape Province, South Africa | | 1918 |
| **H96** | PI 51210 | Mahmoudi | Tunisia | | 1920 |
| **H97** | PI 519380 | BD 1645 | Tunisia | | 1987 |
| **H98** | PI 519751 | D 31729-2L-OL | Federal District, Mexico | | 1987 |
| **H99** | PI 519752 | D 31648-2L-OL | Federal District, Mexico | | 1987 |
| **H100** | PI 519759 | D 73121 | Brazil | | 1987 |
| **H101** | PI 519761 | Maghrebi‘S’ | Federal District, Mexico | | 1987 |
| **H102** | PI 519866 | CB 088 | Federal District, Mexico | | 1987 |
| **H103** | PI 520053 | 31814-1L-OC | Federal District, Mexico | | 1987 |
| **H104** | PI 520173 | Tal | Mexico | | 1987 |
| **H105** | PI 520415 | Syrian Durum 27 | Syria | | 1987 |
| **H106** | PI 532119 | 2515 | Minufiya, Egypt | | 1988 |
| **H107** | PI 546060 | DT367 | Saskatchewan, Canada | | 1990 |
| **H108** | PI 546362 | DT369 | Saskatchewan, Canada | | 1991 |
| **H109** | PI 546462 | Gergana | Khaskovo, Bulgaria | | 1990 |
| **H110** | PI 560335 | KS91WGRC14 | Kansas, United States | | 1992 |
| **H111** | PI 560702 | TU85-008-10-2 | Siirt, Turkey | | 1986 |
| **H112** | PI 560717 | TU85-054-01-2 | Bitlis, Turkey | | 1986 |
| **H113** | PI 560718 | TU85-054-02 | Bitlis, Turkey | | 1986 |
| **H114** | PI 560889 | TU86-24-02-2 | Siirt, Turkey | | 1989 |
| **H115** | PI 56233 | CItr 7041 | Lisboa, Portugal | | 1923 |
| **H116** | PI 565259 | Yurac Mexico | Cochabamba, Bolivia | | 1991 |
| **H117** | PI 565266 | Mexico | Cochabamba, Bolivia | | 1991 |
| **H118** | PI 573005 | Imperial | Arizona, United States | | 1988 |
| **H119** | PI 583724 | 8682-D051-NG | Saskatchewan, Canada | | 1994 |
| **H120** | PI 583731 | G8973-AG1-G | Saskatchewan, Canada | | 1994 |
| **H121** | PI 583732 | G8973-AG1-NG | Saskatchewan, Canada | | 1994 |
| **H122** | PI 583733 | G8973-AQ1-G | Saskatchewan, Canada | | 1994 |
| **H123** | PI 591959 | DW 1 | Cyprus | | 1994 |
| **H124** | PI 593005 | V. 433 | Latium, Italy | | 1996 |
| **H125** | PI 600931 | D-5003 | California, United States | | 1982 |
| **H126** | PI 601250 | Westbred Laker | Arizona, United States | | 1985 |
| **H127** | PI 60712 | Gawi | Egypt | | 1924 |
| **H128** | PI 60742 | Sinai No. 8 | Sinai, Egypt | | 1924 |
| **H129** | PI 610765 | CIGM91.347-6 | Federal District, Mexico | | 1999 |
| **H130** | PI 61112 | CItr 7395 | Kazakhstan | | 1924 |
| **H131** | PI 61123 | CItr 7406 | Kazakhstan | | 1924 |
| **H132** | PI 61189 | CItr 7472 | Krasnoyarsk, Russian Federation | | 1924 |
| **H133** | PI 61351 | Medea | Hokkaido, Japan | | 1924 |
| **H134** | PI 61352 | Roumania | Hokkaido, Japan | | 1924 |
| **H135** | PI 634315 | Canelo | Federal District, Mexico | | 2001 |
| **H136** | PI 634318 | Afuwan | Federal District, Mexico | | 2001 |
| **H137** | PI 656793 | NSGC 19376 | California, United States | | 2009 |
| **H138** | PI 656794 | IR51-8 | California, United States | | 2009 |
| **H139** | PI 656795 | IR17-47 | California, United States | | 2009 |
| **H140** | PI 67341 | Huguenot | Western Australia, Australia | | 1926 |
| **H141** | PI 7016 | Mishriki | Alexandria, Egypt | | 1901 |
| **H142** | PI 70658 | Tulatai Maitai | Heilongjiang, China | | 1926 |
| **H143** | PI 70662 | Lumanian | Heilongjiang, China | | 1926 |
| **H144** | PI 70736 | ICARDA-IG-82459 | Iraq | | 1926 |
| **H145** | PI 7422 | Girgeh | Sawhaj, Egypt | | 1901 |
| **H146** | PI 74830 | ICARDA-IG-82496 | Jiangsu, China | | 1927 |
| **H147** | PI 79900 | N-85 | Heilongjiang, China | | 1929 |
| **H148** | PI 91956 | Chumpe Negro | Junin, Peru | | 1931 |
| **H149** | PI 92024 | Candeal | Cajamarca, Peru | | 1931 |
| **H150** | PI 9872 | Galgalos | Erevan, Armenia | | 1903 |
