# Supplementary material for: Association of Agronomic Traits with SNP Markers in Durum Wheat (Triticum turgidum L. durum (Desf.))
Source: PLoS One. 2015 Jun 25;10(6):e0130854. doi: 10.1371/journal.pone.0130854 (PMC4482485; doi:10.1371/journal.pone.0130854)
Supplement: S2 Table — a PH, plant height; ES, number of effective spikes, LMS, length of main spike; RLMS, rachis internode length of main spike; LFPMS, pillow neck length of main spike; SMS, spikelets on main spike; NSPP, number of spikelets per plant; GNP, Grain number per plant; GWP, grain weight per plant; KGW, 1000-grain weight; b P: the permutation based test for marker significance of individual markers; c R2: the fraction of the total variation explained by the marker after fitting the other model effects. (DOCX) [file pone.0130854.s002.docx]

**S2 Table.** **Significant trait-SNP marker pairs in four consecutive years.**

| **Trait ^a^** | **SNP marker** | **Chromosome location/ bin** | **2010** | | **2011** | | **2012** | | **2013** | |
| --- | --- | --- | --- | --- | --- | --- | --- | --- | --- | --- |
|  |  |  | **P ^b^** | **R^2 c^** | **P ^b^** | **R^2 c^** | **P ^b^** | **R^2 c^** | **P ^b^** | **R^2 c^** |
| **GNP** | AY244508_5_B_Y_26 | 5B | 0.001 | 0.116 | -- | -- | -- | -- | -- | -- |
| **GNP** | BE403637_4_A_Y_452 | 4A | -- | -- | -- | -- | 0.0052 | 0.0785 | -- | -- |
| **GNP** | BE403828__A_N_687 | A | -- | -- | -- | -- | 0.0077 | 0.0525 | -- | -- |
| **GNP** | BE406351_2_A_76 | 2A | 0.0056 | 0.0946 | -- | -- | -- | -- | -- | -- |
| **GNP** | BE406351_2_A_Y_124 | 2A | 0.0056 | 0.0946 | -- | -- | -- | -- | -- | -- |
| **GNP** | BE438226_4_B_Y_286 | 4BL1-0.71-0.86 | -- | -- | -- | -- | -- | -- | 0.0051 | 0.0974 |
| **GNP** | BE442750_4_B_Y_246 | 4B | 0.0078 | 0.0724 | -- | -- | -- | -- | -- | -- |
| **GNP** | BE443538_5_A_1436 | 5AS1-0.40-0.75 | -- | -- | -- | -- | 0.0044 | 0.0602 | -- | -- |
| **GNP** | BE443540_7_B_N_1397 | C-7BL2-0.33 | -- | -- | -- | -- | 0.0074 | 0.0898 | -- | -- |
| **GNP** | BE444562_5_B_286 | C-5BL6-0.29 | -- | -- | 0.0098 | 0.0695 | -- | -- | -- | -- |
| **GNP** | BE444579_3_B_Y_375 | 3B | 0.0083 | 0.0715 | -- | -- | -- | -- | -- | -- |
| **GNP** | BE444864_3_B_373 | 3BL7-0.63-1.00 | 0.0073 | 0.0902 | -- | -- | -- | -- | -- | -- |
| **GNP** | BE445348_3_B_Y_456 | 3B | 0.0049 | 0.0795 | -- | -- | -- | -- | -- | -- |
| **GNP** | BE488206_6_B_Y_206 | 6BS5-0.76-1.05 | 0.0063 | 0.0927 | -- | -- | 0.0057 | 0.0942 | -- | -- |
| **GNP** | BE489692_1_B_Y_171 | 1BL1-0.47-0.69 | 0.008 | 0.0887 | -- | -- | -- | -- | -- | -- |
| **GNP** | BE490226_6_A_Y_308 | 6AL4-0.55-0.90 | 0.0077 | 0.0894 | -- | -- | -- | -- | -- | -- |
| **GNP** | BE490763_2_A_1462 | 2AL1-0.85-1.00 | -- | -- | 0.0098 | 0.0696 | -- | -- | -- | -- |
| **GNP** | BE497494_2_A_Y_475 | 2AS5-0.78-1.00 | -- | -- | -- | -- | -- | -- | 0.0017 | 0.097 |
| **GNP** | BE498418_7_A_148 | C-7AL1-0.39 | 0.0012 | 0.1019 | -- | -- | -- | -- | -- | -- |
| **GNP** | BE499309_3_B_Y_332 | 3BS8-0.78-1.00 | 0.0061 | 0.0764 | -- | -- | -- | -- | -- | -- |
| **GNP** | BE518255_4_A_N_99 | 4BL5-0.86-1.00 | -- | -- | -- | -- | 0.0044 | 0.0812 | -- | -- |
| **GNP** | BE586140_1_A_Y_220 | 1AS3-0.86-1.00 | 0.0047 | 0.0804 | -- | -- | -- | -- | 0.0084 | 0.0721 |
| **GNP** | BE590521_6_B_N_331 | C-6BL3-0.36 | -- | -- | -- | -- | 0.0044 | 0.0602 | -- | -- |
| **GNP** | BE590553_7_A_190 | 7AS5-0.59-0.89 | 0.0056 | 0.0777 | -- | -- | -- | -- | -- | -- |
| **GNP** | BE590634_1_B_338 | 1BL1-0.47-1.00* | -- | -- | 0.0063 | 0.0936 | -- | -- | -- | -- |
| **GNP** | BE591243_2_A_109 | C-2AL1-0.85 | 0.0099 | 0.0688 | -- | -- | -- | -- | -- | -- |
| **GNP** | BE591739_4_A_Y_622 | 4A | 0.006 | 0.0766 | -- | -- | -- | -- | -- | -- |
| **GNP** | BE591861_4_A_Y_186 | C-4AS1-0.20 | 0.0078 | 0.0724 | -- | -- | -- | -- | -- | -- |
| **GNP** | BE604119_6_B_733 | C-6BS5-0.76 | 0.0026 | 0.0897 | -- | -- | -- | -- | -- | -- |
| **GNP** | BE605063_1_A_Y_252 | 1A | 0.0093 | 0.0862 | -- | -- | -- | -- | 0.0073 | 0.0914 |
| **GNP** | BE637838_7_A_Y_574 | 7AL16-0.86-0.90 | -- | -- | -- | -- | 0.0066 | 0.0918 | -- | -- |
| **GNP** | BF202706_4_A_Y_466 | C-4AL12-0.43 | 0.0013 | 0.1002 | -- | -- | -- | -- | -- | -- |
| **GNP** | BF291928_3_B_38 | 3BL10-0.50-0.63 | -- | -- | -- | -- | -- | -- | 0.0095 | 0.0703 |
| **GNP** | BF293541_4_A_Y_88 | 4AL5-0.66-0.80 | -- | -- | 0.0042 | 0.1003 | -- | -- | -- | -- |
| **GNP** | BF428573_6_A_Y_195 | 6AL4-0.55-0.90 | 0.0078 | 0.0724 | -- | -- | -- | -- | -- | -- |
| **GNP** | BF428701_6_B_287 | 6BS | 0.0049 | 0.0799 | -- | -- | -- | -- | -- | -- |
| **GNP** | BF428701_6_B_Y_447 | 6BS | 0.0037 | 0.0841 | -- | -- | -- | -- | -- | -- |
| **GNP** | BF429186_1_B_21 | 1B | -- | -- | -- | -- | -- | -- | 0.0001 | 0.1378 |
| **GNP** | BF474340_1_B_N_65 | 1BL1-0.47-0.69 | 0.0098 | 0.0689 | -- | -- | -- | -- | -- | -- |
| **GNP** | BF483091_6_A_Y_472 | 6AL8-0.90-1.00 | -- | -- | -- | -- | -- | -- | 0.0032 | 0.0873 |
| **GNP** | BF483362_5_A_Y_160 | C-5AL12-0.35 | 0.0084 | 0.0713 | -- | -- | -- | -- | -- | -- |
| **GNP** | BG262734_3_A_N_190 | 3AL5-0.78-1.00 | 0.0097 | 0.069 | -- | -- | -- | -- | -- | -- |
| **GNP** | BG263521_2_A_61 | C-2AS5-0.78 | 0.0075 | 0.0898 | -- | -- | -- | -- | -- | -- |
| **GNP** | BG274742_6_A_N_827 | C-6AS1-0.35 | -- | -- | -- | -- | 0.002 | 0.0711 | -- | -- |
| **GNP** | BG314205_1_B_33 | C-1BL6-0.32 | -- | -- | -- | -- | 0.0034 | 0.0639 | -- | -- |
| **GNP** | BG608354_2_A_Y_100 | 2AS5-0.78-1.00 | -- | -- | 0.0073 | 0.091 | -- | -- | -- | -- |
| **GNP** | BM137927_3_A_Y_157 | 3AL5-0.78-1.00 | 0.0086 | 0.0709 | -- | -- | -- | -- | -- | -- |
| **GNP** | BQ161465_2_B_Y_242 | 2B | 0.0044 | 0.0813 | -- | -- | -- | -- | -- | -- |
| **GNP** | BQ169999_5_B_Y_301 | 5B | 0.0059 | 0.0564 | -- | -- | -- | -- | 0.008 | 0.0527 |
| **GNP** | CD452629_7_B_Y_270 | 7B | 0.0024 | 0.0689 | -- | -- | -- | -- | -- | -- |
| **GNP** | CD452643_6_B_111 | 6B | -- | -- | -- | -- | -- | -- | 0.0042 | 0.083 |
| **GNP** | CD453913_7_A_105 | 7A | 0.0063 | 0.0928 | -- | -- | -- | -- | -- | -- |
| **GNP** | CD454152_5_B_140 | 5B | -- | -- | -- | -- | -- | -- | 0.0058 | 0.078 |
| **GNP** | CD454870_7_A_Y_228 | 7A | 0.005 | 0.0795 | -- | -- | -- | -- | -- | -- |
| **GWP** | BE403422_6_A_Y_119 | C-6AL4-0.55 | -- | -- | -- | -- | 0.0096 | 0.0693 | -- | -- |
| **GWP** | BE405834_1_B_Y_216 | 1B | -- | -- | -- | -- | 0.0057 | 0.0568 | -- | -- |
| **GWP** | BE443538_5_A_1436 | 5AS1-0.40-0.75 | -- | -- | -- | -- | 0.0043 | 0.0608 | -- | -- |
| **GWP** | BE446310_3_B_Y_167 | 3B | -- | -- | -- | -- | 0.0083 | 0.088 | -- | -- |
| **GWP** | BE490763_2_A_1462 | 2AL1-0.85-1.00 | -- | -- | 0.0079 | 0.0724 | -- | -- | -- | -- |
| **GWP** | BE497494_2_A_Y_475 | 2AS5-0.78-1.00 | -- | -- | -- | -- | -- | -- | 0.0065 | 0.0762 |
| **GWP** | BE500634_4_A_Y_118 | 4AL12-0.43-0.59 | -- | -- | 0.0065 | 0.0551 | -- | -- | -- | -- |
| **GWP** | BE590521_6_B_N_331 | C-6BL3-0.36 | -- | -- | -- | -- | 0.0043 | 0.0608 | -- | -- |
| **GWP** | BE606541_6_B_Y_676 | 6B | -- | -- | -- | -- | 0.0096 | 0.0692 | -- | -- |
| **GWP** | BE637864_1_A_Y_141 | 1AS1-0.47-0.86 | -- | -- | 0.0065 | 0.0551 | -- | -- | -- | -- |
| **GWP** | BF200773_6_B_Y_533 | 6BL5-0.40-1.00 | -- | -- | 0.0065 | 0.0551 | -- | -- | -- | -- |
| **GWP** | BF429186_1_B_21 | 1B | -- | -- | -- | -- | -- | -- | 0.0009 | 0.1087 |
| **GWP** | BF473825_7_B_Y_293 | 7BS1-0.27-1.00 | -- | -- | 0.0065 | 0.0551 | -- | -- | -- | -- |
| **GWP** | BF474139_1_A_144 | 1AL3-0.61-1.00 | -- | -- | -- | -- | -- | -- | 0.0038 | 0.0846 |
| **GWP** | BF483091_6_A_Y_472 | 6AL8-0.90-1.00 | -- | -- | -- | -- | -- | -- | 0.0014 | 0.1005 |
| **GWP** | BG314205_1_B_33 | C-1BL6-0.32 | -- | -- | -- | -- | 0.0056 | 0.0571 | -- | -- |
| **GWP** | BQ161779_6_A_631 | 6A | -- | -- | 0.0065 | 0.0551 | -- | -- | -- | -- |
| **GWP** | CD452643_6_B_111 | 6B | -- | -- | -- | -- | -- | -- | 0.0092 | 0.0709 |
| **NSPP** | BE399999_1_A_172 | 1AL1-0.17-0.61 | -- | -- | -- | -- | -- | -- | 0.008 | 0.0524 |
| **NSPP** | BE405849_5_A_Y_179 | C-5AL10-0.57* | -- | -- | -- | -- | -- | -- | 0.0087 | 0.0711 |
| **NSPP** | BE442676_5_A_Y_220 | 5AL12-0.35-0.57 | -- | -- | -- | -- | -- | -- | 0.0067 | 0.0923 |
| **NSPP** | BE443332_1_B_97 | 1BL1-0.47-0.69 | -- | -- | -- | -- | -- | -- | 0.0009 | 0.1064 |
| **NSPP** | BE445506_7_A_Y_818 | 7AL18-0.90-1.00 | -- | -- | 0.0013 | 0.1123 | -- | -- | -- | -- |
| **NSPP** | BE489692_1_B_Y_91 | 1BL1-0.47-0.69 | -- | -- | -- | -- | -- | -- | 0.0056 | 0.0952 |
| **NSPP** | BE495790_5_A_N_802 | 5AL12-0.35-0.57 | -- | -- | -- | -- | -- | -- | 0.001 | 0.1052 |
| **NSPP** | BE498323_7_A_Y_331 | 7AL1-0.39-0.71 | -- | -- | -- | -- | -- | -- | 0.0006 | 0.0902 |
| **NSPP** | BE590553_7_A_190 | 7AS5-0.59-0.89 | -- | -- | -- | -- | -- | -- | 0.0087 | 0.0712 |
| **NSPP** | BE606541_6_B_Y_566 | 6B | -- | -- | -- | -- | -- | -- | 0.0031 | 0.0875 |
| **NSPP** | BE636954_5_A_Y_385 | 5AS7/10-0.98-1.00 | -- | -- | -- | -- | 0.0087 | 0.0878 | -- | -- |
| **NSPP** | BF482950_4_A_Y_272 | 4A | -- | -- | -- | -- | 0.0071 | 0.0912 | -- | -- |
| **NSPP** | BG274584_2_A_Y_495 | C-2AL1-0.85 | -- | -- | -- | -- | -- | -- | 0.0061 | 0.0562 |
| **NSPP** | BQ169669_7_B_Y_379 | 7B | -- | -- | -- | -- | -- | -- | 0.0012 | 0.103 |
| **NSPP** | BQ169999_5_B_Y_301 | 5B | -- | -- | -- | -- | -- | -- | 0.0014 | 0.0769 |
| **NSPP** | CD453912_6_B_53 | 6B | -- | -- | -- | -- | -- | -- | 0.0006 | 0.1147 |
| **LFPMS** | CD454152_5_B_76 | 5B | 0.0059 | 0.0902 | -- | -- | -- | -- | -- | -- |
| **KGW** | AY244508_5_B_Y_26 | 5B | -- | -- | -- | -- | 0.001 | 0.111 | -- | -- |
| **KGW** | BE425301_4_A_Y_160 | 4AS4-0.63-0.76 | 0.003 | 0.0662 | -- | -- | -- | -- | -- | -- |
| **KGW** | BE445667_6_B_Y_285 | C-6BS5-0.76 | -- | -- | -- | -- | 0.0025 | 0.0689 | -- | -- |
| **KGW** | BE495786_1_B_108 | 1BL1-0.47-0.69 | -- | -- | -- | -- | 0.0024 | 0.0694 | -- | -- |
| **KGW** | BE637476_7_B_N_544 | 7BL10-0.78-1.00 | -- | -- | -- | -- | 0.002 | 0.0945 | -- | -- |
| **KGW** | BG605368_2_A_Y_310 | C-2AL1-0.85 | -- | -- | -- | -- | 0.005 | 0.0971 | -- | -- |
| **KGW** | CD454448_6_A_84 | 6A | 0.0071 | 0.054 | -- | -- | -- | -- | -- | -- |
| **RLMS** | BE443538_5_A_1436 | 5AS1-0.40-0.75 | -- | -- | -- | -- | 0.0045 | 0.0594 | -- | -- |
| **RLMS** | BE590521_6_B_N_331 | C-6BL3-0.36 | -- | -- | -- | -- | 0.0045 | 0.0594 | -- | -- |
| **RLMS** | BF475120_6_B_Y_75 | 6BL5-0.40-1.00 | -- | -- | -- | -- | -- | -- | 0.0055 | 0.0581 |
| **RLMS** | BF483091_6_A_Y_472 | 6AL8-0.90-1.00 | 0.0094 | 0.0795 | -- | -- | -- | -- | -- | -- |
| **RLMS** | BG314205_1_B_33 | C-1BL6-0.32 | -- | -- | -- | -- | 0.0053 | 0.0572 | -- | -- |
| **ES** | BE399999_1_A_172 | 1AL1-0.17-0.61 | -- | -- | -- | -- | -- | -- | 0.0032 | 0.0653 |
| **ES** | BE403154_6_A_Y_45 | 6AL8-0.90-1.00 | -- | -- | -- | -- | -- | -- | 0.0082 | 0.0888 |
| **ES** | BE404841_3_A_N_272 | 3A | -- | -- | 0.0076 | 0.0834 | -- | -- | -- | -- |
| **ES** | BE443332_1_B_97 | 1BL1-0.47-0.69 | -- | -- | -- | -- | -- | -- | 0.0036 | 0.0852 |
| **ES** | BE489692_1_B_Y_91 | 1BL1-0.47-0.69 | -- | -- | -- | -- | -- | -- | 0.0094 | 0.0864 |
| **ES** | BE495790_5_A_N_802 | 5AL12-0.35-0.57 | -- | -- | -- | -- | -- | -- | 0.0037 | 0.0848 |
| **ES** | BE498323_7_A_Y_331 | 7AL1-0.39-0.71 | -- | -- | -- | -- | -- | -- | 0.0019 | 0.0727 |
| **ES** | BE606541_6_B_Y_566 | 6B | -- | -- | -- | -- | -- | -- | 0.007 | 0.0746 |
| **ES** | BG274584_2_A_Y_495 | C-2AL1-0.85 | -- | -- | -- | -- | -- | -- | 0.001 | 0.0814 |
| **ES** | BQ169669_7_B_Y_379 | 7B | -- | -- | -- | -- | -- | -- | 0.0034 | 0.0858 |
| **ES** | BQ169999_5_B_Y_301 | 5B | -- | -- | -- | -- | -- | -- | 0.0004 | 0.0971 |
| **ES** | BQ171683_7_B_256 | 7B | -- | -- | -- | -- | -- | -- | 0.009 | 0.051 |
| **ES** | CD453912_6_B_53 | 6B | -- | -- | -- | -- | -- | -- | 0.0001 | 0.1446 |
| **PH** | BF475120_6_B_Y_75 | 6BL5-0.40-1.00 | 0.001 | 0.0946 | 0.0024 | 0.0728 | 0.0019 | 0.0732 | 0.0017 | 0.0748 |
| **PH** | BE405269_4_B_84 | 4B | 0.0042 | 0.0704 | 0.0065 | 0.0583 | 0.0069 | 0.0547 | 0.0077 | 0.0533 |
| **PH** | BE443948_2_A_Y_345 | C-2AL1-0.85 | -- | -- | 0.0093 | 0.0738 | 0.0094 | 0.0703 | -- | -- |
| **PH** | BF475120_6_B_67 | 6BL5-0.40-1.00 | 0.0038 | 0.0966 | 0.0099 | 0.0728 | 0.0073 | 0.0742 | 0.0069 | 0.0754 |
| **PH** | BE490041_1_A_371 | 1AS3-0.86-1.00 | -- | -- | -- | -- | 0.0067 | 0.0756 | 0.0061 | 0.0773 |
| **PH** | BG312827_6_A_Y_305 | 6A | 0.0026 | 0.0781 | -- | -- | 0.0082 | 0.0523 | 0.0079 | 0.053 |
| **SMS** | AJ612027_5_A_Y_238 | 5A | -- | -- | -- | -- | -- | -- | 0.0073 | 0.0743 |
| **SMS** | AJ612027_5_A_Y_473 | 5A | -- | -- | -- | -- | -- | -- | 0.0073 | 0.0743 |
| **SMS** | BE404341_5_A_Y_258 | C-5AL12-0.35 | -- | -- | -- | -- | -- | -- | 0.001 | 0.1066 |
| **SMS** | BE404717_4_A_Y_280 | 4AS4-0.63-0.76 | -- | -- | 0.006 | 0.0776 | -- | -- | -- | -- |
| **SMS** | BE426620_2_A_Y_420 | 2AL1-0.85-1.00 | 0.005 | 0.0978 | -- | -- | -- | -- | -- | -- |
| **SMS** | BE443948_2_A_Y_345 | C-2AL1-0.85 | 0.0095 | 0.0703 | -- | -- | -- | -- | -- | -- |
| **SMS** | BE444144_2_B_92 | 2BS | -- | -- | -- | -- | -- | -- | 0.0049 | 0.0806 |
| **SMS** | BE444144_2_B_N_138 | 2BS | -- | -- | -- | -- | -- | -- | 0.0095 | 0.0503 |
| **SMS** | BE445278_2_B_143 | 2B | -- | -- | -- | -- | 0.0031 | 0.0892 | -- | -- |
| **SMS** | BE445506_7_B_Y_152 | 7BL10-0.78-1.00 | -- | -- | -- | -- | -- | -- | 0.0004 | 0.1399 |
| **SMS** | BE495175_3_A_Y_317 | C-3AL3-0.42 | 0.0068 | 0.0551 | -- | -- | -- | -- | -- | -- |
| **SMS** | BE495277_5_A_Y_427 | C-5AL10-0.57* | -- | -- | 0.0022 | 0.0935 | -- | -- | -- | -- |
| **SMS** | BE585797_3_A_Y_283 | 3AL3-0.42-0.78 | 0.0058 | 0.0952 | -- | -- | -- | -- | -- | -- |
| **SMS** | BE590553_7_A_190 | 7AS5-0.59-0.89 | -- | -- | -- | -- | 0.0045 | 0.0833 | -- | -- |
| **SMS** | BF200891_7_B_80 | 7BS1-0.27-1.00 | 0.0075 | 0.0741 | -- | -- | -- | -- | -- | -- |
| **SMS** | BF293620_5_A_Y_246 | 5A | -- | -- | -- | -- | -- | -- | 0.001 | 0.1066 |
| **SMS** | BF485380_7_B_Y_375 | 7B | -- | -- | -- | -- | -- | -- | 0.0061 | 0.0771 |
| **SMS** | BG274294_1_B_382 | 1B | -- | -- | 0.0073 | 0.0542 | -- | -- | -- | -- |
| **SMS** | BG314551_3_A_Y_162 | 3AS4-0.45-1.00 | 0.0047 | 0.0812 | -- | -- | 0.0042 | 0.0846 | 0.0041 | 0.0834 |
| **SMS** | BQ166748_6_A_N_149 | 6A | 0.0045 | 0.0609 | -- | -- | -- | -- | -- | -- |
| **SMS** | BQ168780_5_B_995 | 5B | -- | -- | -- | -- | 0.0064 | 0.0778 | -- | -- |
| **SMS** | BQ169501_7_A_Y_296 | 7A | 0.0063 | 0.0766 | -- | -- | -- | -- | -- | -- |
| **LMS** | BF474284_1_B_Y_357 | 1BL2-0.69-0.85 | 0.0025 | 0.0899 | 0.0003 | 0.126 | 0.0042 | 0.0865 | 0 | 0.2609 |
| **LMS** | BG605368_2_A_Y_310 | C-2AL1-0.85 | 0 | 0.1824 | 0.0008 | 0.1309 | 0.0024 | 0.115 | 0.0025 | 0.1087 |
| **LMS** | BM134437_3_A_Y_233 | 3AL5-0.78-1.00 | 0.0005 | 0.1166 | 0.0011 | 0.105 | 0.0007 | 0.1165 | 0.0002 | 0.1285 |
| **LMS** | BE445667_6_B_Y_285 | C-6BS5-0.76 | 0.0001 | 0.1204 | 0.0056 | 0.0582 | 0.0023 | 0.0732 | 0.0001 | 0.1103 |
| **LMS** | BF474615_4_A_98 | 4AL13-0.59-0.66 | -- | -- | 0.0067 | 0.0763 | -- | -- | -- | -- |
| **LMS** | BF484028_5_A_Y_97 | 5AL10-0.57-0.78 | 0.0092 | 0.0699 | 0.0089 | 0.0718 | -- | -- | -- | -- |
| **LMS** | BE443538_5_A_Y_547 | 5AS1-0.40-0.75 | -- | -- | -- | -- | -- | -- | 0.0008 | 0.0849 |
| **LMS** | BE444359_1_B_270 | 1BL2-0.69-0.85 | -- | -- | -- | -- | -- | -- | 0.0073 | 0.074 |
| **LMS** | BE495786_1_B_108 | 1BL1-0.47-0.69 | 0.0063 | 0.0554 | -- | -- | 0.0063 | 0.0584 | -- | -- |
| **LMS** | BE496826_6_A_Y_402 | 6AS5-0.65-1.00 | -- | -- | -- | -- | 0.0019 | 0.0992 | -- | -- |
| **LMS** | BE591974_5_A_Y_142 | 5AS1-0.40-0.75 | -- | -- | -- | -- | -- | -- | 0.0044 | 0.0609 |
| **LMS** | BF291774_6_A_Y_481 | 6A | 0.0017 | 0.0741 | -- | -- | 0.0084 | 0.0543 | -- | -- |
| **LMS** | BF291774_6_B_181 | 6B | 0.0017 | 0.0741 | -- | -- | 0.0084 | 0.0543 | -- | -- |
| **LMS** | BF291774_6_B_519 | 6B | 0.007 | 0.0741 | -- | -- | -- | -- | -- | -- |
| **LMS** | BF292264_7_A_712 | 7AS1-0.89-1.00 | -- | -- | -- | -- | -- | -- | 0.0005 | 0.1155 |
| **LMS** | BF292614_6_B_427 | 6BL5-0.40-1.00 | -- | -- | -- | -- | 0.0023 | 0.073 | 0.0002 | 0.1048 |
| **LMS** | BG262882_1_B_217 | 1BL3-0.85-1.00 | -- | -- | -- | -- | -- | -- | 0.0024 | 0.069 |
| **LMS** | BG262882_1_B_94 | 1BL3-0.85-1.00 | -- | -- | -- | -- | -- | -- | 0.0024 | 0.069 |
| **LMS** | BG263769_3_A_Y_55 | 3A | -- | -- | -- | -- | -- | -- | 0.0035 | 0.0854 |
| **LMS** | BG313707_5_A_Y_547 | 5AS1-0.40-0.75 | -- | -- | -- | -- | -- | -- | 0.0008 | 0.0849 |
| **LMS** | BG605368_2_A_156 | C-2AL1-0.85 | 0.0081 | 0.0883 | -- | -- | -- | -- | -- | -- |
| **LMS** | BQ159615_6_B_Y_336 | 6B | -- | -- | -- | -- | -- | -- | 0.0048 | 0.0806 |
| **LMS** | BQ169448_6_B_252 | 6B | -- | -- | -- | -- | -- | -- | 0.0052 | 0.0792 |

^a^ PH, plant height; ES, number of effective spikes, LMS, length of main spike; RLMS, rachis internode length of main spike; LFPMS, pillow neck length of main spike; SMS, spikelets on main spike; NSPP, number of spikelets per plant; GNP, Grain number per plant; GWP, grain weight per plant; KGW, 1000-grain weight.

^b^ P: the permutation based test for marker significance of individual markers.

^c^ R^2^: the fraction of the total variation explained by the marker after fitting the other model effects.
